# Synergistic Optimization of Buried Interface by Multifunctional Organic–Inorganic Complexes for Highly Efficient Planar Perovskite Solar Cells

**DOI:** 10.1007/s40820-023-01130-5

**Published:** 2023-06-19

**Authors:** Heng Liu, Zhengyu Lu, Weihai Zhang, Hongkang Zhou, Yu Xia, Yueqing Shi, Junwei Wang, Rui Chen, Haiping Xia, Hsing-Lin Wang

**Affiliations:** 1 School of Materials Science and Engineering, Harbin Institute of Technology, Harbin, 150001 People’s Republic of China; 2 Department of Materials Science and Engineering, Southern University of Science and Technology, Shenzhen, 518055 Guangdong People’s Republic of China; 3 Shenzhen Grubbs Institute and Department of Chemistry, Southern University of Science and Technology, Shenzhen, 518055 Guangdong People’s Republic of China; 4 Department of Electrical and Electronic Engineering, Southern University of Science and Technology, Shenzhen, 518055 Guangdong People’s Republic of China; 5 Key University Laboratory of Highly Efficient Utilization of Solar Energy and Sustainable Development of Guangdong, Southern University of Science and Technology, Shenzhen, 518055 Guangdong People’s Republic of China

**Keywords:** Perovskite solar cells, Organic, Inorganic complexes, Multifunctional interfacial material, Buried interface layer

## Abstract

For the further improvement of the power conversion efficiency (PCE) and stability of perovskite solar cells (PSCs), the buried interface between the perovskite and the electron transport layer is crucial. However, it is challenging to effectively optimize this interface as it is buried beneath the perovskite film. Herein, we have designed and synthesized a series of multifunctional organic–inorganic (OI) complexes as buried interfacial material to promote electron extraction, as well as the crystal growth of the perovskite. The OI complex with BF_4_^−^ group not only eliminates oxygen vacancies on the SnO_2_ surface but also balances energy level alignment between SnO_2_ and perovskite, providing a favorable environment for charge carrier extraction. Moreover, OI complex with amine (− NH_2_) functional group can regulate the crystallization of the perovskite film via interaction with PbI_2_, resulting in highly crystallized perovskite film with large grains and low defect density. Consequently, with rational molecular design, the PSCs with optimal OI complex buried interface layer which contains both BF_4_^−^ and −NH_2_ functional groups yield a champion device efficiency of 23.69%. More importantly, the resulting unencapsulated device performs excellent ambient stability, maintaining over 90% of its initial efficiency after 2000 h storage, and excellent light stability of 91.5% remaining PCE in the maximum power point tracking measurement (under continuous 100 mW cm^−2^ light illumination in N_2_ atmosphere) after 500 h.

**Supplementary Information:**

The online version contains supplementary material available at 10.1007/s40820-023-01130-5.

## Highlights

- Highly performed perovskite solar cells are achieved via introducing organic–inorganic CL–NH complex as multifunctional interfacial layer.
- CL–NH complex not only reduces oxygen vacancies on the surface of SnO_2_ but also regulates film crystallization, resulting in a superior device efficiency of 23.69%.
- The resulting device performs excellent stability with 91.5% initial power conversion efficiency retained after 500 h light illumination.

**Supplementary Information:**

The online version contains supplementary material available at 10.1007/s40820-023-01130-5.

## Graphical abstract

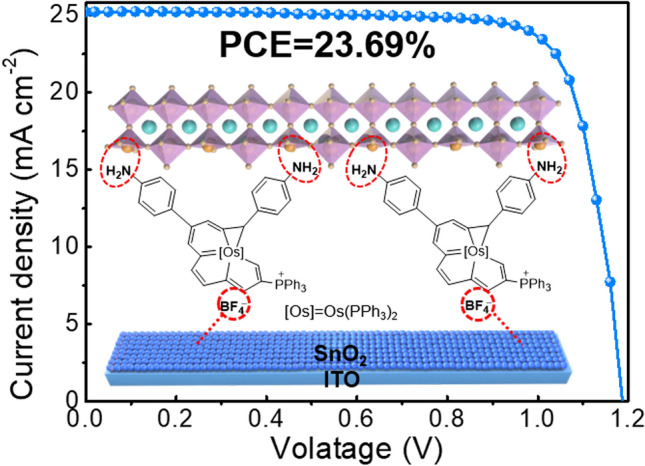

## Introduction

Organic–inorganic hybrid perovskite solar cells (OIHP) have received extensive attention in the past decade due to their low fabrication cost and high photovoltaic performance [1-4]. To date, a certified power conversion efficiency (PCE) of 25.7% [5], which is comparable to that of commercialized single crystal silicon solar cells, has been achieved, showing great potential toward practical application. At present, one of the most commonly used electron transport layers (ETL) used in perovskite solar cells (PSCs) is tin oxide (SnO_2_) [6, 7]. Compared to its counterparts, such as titanium dioxide (TiO_2_) [8] and zinc oxide (ZnO) [9], SnO_2_ exhibits higher electron mobility and better energy level alignment matching with perovskite films, contributing to superior device performance, whereas the presence of considerable amount of oxygen vacancies on the SnO_2_ surface would act as traps to capture the charge carriers, which inevitably induce non-radiative recombination at the SnO_2_/perovskite interface, and thus deteriorating device performance [10-13].

To overcome these issues, various effective methods including the introduction of alkali metal cations (such as Li^+^, Na^+^, K^+^, Rb^+^, and Cs^+^) and functional anions (for example, CH_3_COO^−^, Cl^−^, PF_6_^−^ and BF_4_^−^) have been developed [14, 15]. Particularly, the introduction of functional BF_4_^−^ anion group to modify charge transport layers (CTLs) has been considered as a promising strategy to reduce the oxygen vacancies on the CTLs surface, thereby promoting charge transport and matching the energy level of the perovskite layer. For instance, Gao et al. incorporated 4-Fluorophenylammonium tetrafluoroborate (FBABF_4_) consisting of simultaneously fluorinated anion and cation at SnO_2_/perovskite interface. The formation of coordination bonds between fluorine atoms and SnO_2_ on BF_4_^−^ can effectively reduce the formation of oxygen vacancies and promote efficient charge transport [16]. Similarly, Zhu et al. introduced an interlayer of phenylethylamine tetrafluoroborate (PEABF_4_) at the ETL/PVK interface. They indicated that the functional PEABF_4_ can passivate the oxygen defects of TiO_2_ and the interface defects of the ETL/PVK, thus accelerating the electron extraction rate and inhibiting carrier non-radiative recombination [17].

Apart from SnO_2_ ETL, the perovskite layer also has large number of structural defects at the perovskite bottom surface, especially for two-step method fabricated perovskite film [18, 19]. These defects act primarily as non-radiative recombination centers, leading to poor device performance. To mitigate the notorious recombination and enhance device performance, researchers have developed buried interface engineering which aimed to passivate the interface defects and balances the energy level mismatch [20]. For instance, Wu et al. employed a large alkylammonium interlayer (LAI) to reduce the energy loss occurred between transport layers and perovskite [21]. They found that the LAIs not only suppress the interfacial non-radiative recombination induced by the surface defects but also increase the charge selectivity and extraction from perovskite to transport layers, contributing to a champion PCE over 22%, which is among the highest efficiencies reported for inverted PSCs. In contrast, Xu et al. adopted CsI-SnO_2_ complex as ETL to fabricate efficient and stable PSCs [22]. They have indicated that the CsI modification facilitates the growth of the perovskite film and effectively passivates the interfacial defects. Meanwhile, the gradient distribution of Cs^+^ contributes to a more suitable band alignment with perovskite. Eventually, a significantly improved PCE up to 23.3% of FAPbI_3_-based PSCs is achieved. In our previous work, 1,8-Octanediamine dihydroiodide (ODADI) was adopted to develop an alkylammonium pre-deposition strategy for the fabrication of high-quality perovskite film. Our results suggest that the pre-deposited ODADI layer not only facilitates perovskite crystallization but also passivates the buried interface defects, resulting in perovskite film with high crystallinity and superior electronic properties [23-25]. Since these studies have demonstrated that buried interface engineering is effective on enhancing device performance, a buried interface layer is expected to be more versatile. For example, in addition to perovskite structural defects passivation, a well-designed buried interface layer with ability to eliminate oxygen vacancies on the SnO_2_ ETL surface can further enhance device performance.

To achieve the above aims, a series of organic–inorganic (OI) complexes (CL–Ph, CL–BPh, CL–NH) were synthesized and introduced as buried interface layer to fabricate high performance two-step method-based PSCs. Our results suggest that OI complex with BF_4_^−^ group can not only eliminate oxygen vacancies on the SnO_2_ surface but also balance energy level alignment between SnO_2_ and perovskite, providing a favorable environment for charge carrier extraction, while OI complex with amine (−NH_2_) functional group can regulate the crystallization of the perovskite film via interaction with PbI_2_, resulting in highly crystallized perovskite film with large grain size and low defect density. Finally, with rationally design, the PSCs with CL–NH buried interface layer which contains both BF_4_^−^ and −NH_2_ functional groups yield a champion device efficiency of 23.69%. Moreover, the resulting unencapsulated device performs excellent ambient stability, maintaining over 90% of its initial efficiency after 2000 h storage, and excellent light stability of 91.5% remaining PCE in the MPP measurement (under continuous 100 mW cm^−2^ light illumination, in N_2_ atmosphere) after 500 h. This multifunctional OI complex provides a new direction toward designing more versatile buried interface layer for highly efficient and stable PSCs.

## Experimental Section

### Materials

ITO glass was purchased from Advanced Election Technology (China). SnO_2_ were purchased from Alfa Aesar. Formamidinium Iodide (FAI), Methylammonium Bromide (MABr), Methylammonium Chloride (MACl), 4-tert-Butylpyridine and Lithium-bis (trifluoromethanesulfonyl) imide (Li-TFSI) were purchased from Advanced Election Technology (China). Spiro-OMeTAD, Lead (II) iodide PbI_2_ were purchased from Xi’an Polymer Light Technology Corp (Xi’an p-OLED). *N*,*N*-dimethylformamide (DMF), dimethylsulfoxide (DMSO), chlorobenzene (CB), isopropyl alcohol (IPA),acetonitrile (ACN) and ethanol purchased from Sigma-Aldrich. Gold (Au, 99.99%) were obtained from commercial sources.

### Solution Preparation and Device Fabrication

#### Solution Preparation

SnO_2_ colloid solution (15 wt%) was diluted within DI water (1:5.5, v: v), and stirring at room temperature for 10 min, followed use a syringe and an aqueous filter to filter the above solution. The preparation of PbI_2_ precursor solution, 599.3 mg PbI_2_ powder was dissolved in 1 mL DMF/DMSO 950:50 and stirred overnight at 70 °C. The preparation of the solution of organic amine salts, an isopropyl alcohol (IPA) solution containing organic salts ( the mass ratio of FAI: MABr: MACl is 60 mg: 6 mg: 6 mg), was stirred at 70 °C for 30 min. The preparation of the solution of Spiro-OMeTAD HTL consisted of 72.3 mg spiro-OMeTAD, 28.8 μL 4-tert-butylpyridine, 17.5 μL lithium-bis (trifluoromethanesulfonyl) imide (Li-TFSI) solution (520 mg Li-TFSI in 1 mL acetonitrile), and 1 mL chlorobenzene. The preparation of organic–inorganic (OI) complexes solution, 1 mg CL–BPh, CL–Ph and CL–NH powder was dissolved in 1 mL DMF and stirred at room temperature.

#### Device Fabrication

The glass/ITO substrate was first scrubbed in detergent and then sequentially cleaned by sonication in deionized water, acetone, and isopropanol for 25 min, respectively. After that, nitrogen dried glass/ITO were treated with plasma for 5 min before usage. Then deposit SnO_2_ on the substrate as an electron transport layer by spin-coated at 3500 rpm for 30 s and annealed in ambient air at 150 °C for 30 min on the hot plate. After cooling to room temperature, the substrates were transferred to a nitrogen filled glove box. Then, the dissolved CL–BPh, CL–Ph and CL–NH are spin-coated on the surface of the SnO_2_ layer at 4000 rpm for 30 s. After that the 1.3 M of PbI_2_ dissolved in anhydrous DMF: DMSO 95:5 (v:v) was spin coated onto SnO_2_ at 2000 rpm for 30 s, and heating at 70 °C for 1 min and cooling 10 min, followed the mixture solution of FAI: MABr: MACl (60 mg:6 mg:6 mg in 1 mL IPA) was spin-coating onto the PbI_2_ at 3000 rpm for 30 s, then transferred into ambient air (RH30%–40%) filled glove box annealed at 150 °C for 10 min on the hot plate. After the substrates were transferred to a nitrogen filled glove box, cooling to room temperature. Subsequently, the substrate transferred into nitrogen filled glove box was deposited on the perovskite films as the hole-transport layer by spin-casting the Spiro-OMeTAD solution at 4000 rpm for 30 s. Finally, an approximate 80 nm thick of Au electrode was fabricated using a shadow mask under high vacuum by thermal evaporation.

### Characterizations

The crystal structure and phase of the perovskite which were characterized using X-ray diffraction (XRD) spectrometer were obtained on Bruker Advanced D8 X-ray diffractometer using Cu Kα (λ = 0.154 nm) radiation. A UV–Vis spectrophotometer (Agilent Cary 5000) was used to collect the absorbance spectra of the perovskite films. Steady-state photoluminescence (PL) spectra were recorded on Shimadzu RF-5301pc. Time-resolved photoluminescence spectra were measured on a PL system (Fluo-Time 300) under excitation with a picosecond pulsed diode laser with a repetition frequency of 1 MHz. The morphology of the films was studied by field-emission scanning electron microscopy (SEM; TESCAM MIRA3). The surface potential of perovskite films obtained with a atomic force microscope (AFM; Asylum Research MFP-3D-Stand Alone). X-ray photoelectron spectroscopy (XPS) was conducted on a Thermo ScientificTM K-AlphaTM + spectrometer equipped with a monochromatic Al Kα X-ray source (1486.6 eV) operating at 100 W. Samples were analyzed under vacuum (*P* < 10^−8^ mbar) with a pass energy of 150 eV (survey scans) or 50 eV (high-resolution scans). The XPS spectra were calibrated by the binding energy of 284.8 eV for C 1*s*. Ultraviolet photoelectron spectroscopy (UPS, ESCALAB 250Xi, Thermo Fisher) measurements were carried out using a He Iα photon source (21.22 eV). The current density–voltage (*J–V*) curves of fabricated devices were obtained from the forward and reverse scan with 30 mV intervals and 10 ms delay time under AM 1.5 G illumination (100 mW cm^−2^) were collected using a source meter (Keysight B2901A) and a solar simulator (Enlitech SS-F5-3A). The EQE spectra were measured using a quantum efficiency measurement system (Enlitech QER-3011) in which the light intensity at every wavelength was calibrated with a Si detector before measurement. The maximum power point (MPP) output was measured by testing the steady-state current density at the maximum power point voltage. Electrochemical impedance spectroscopy (EIS) was tested with the frequency range from 100 Hz to 1 MHz by the electrochemical workstation (Princeton Applied Research, P4000 +) in the dark conditions at with a bias of 1 V. The amplitude is 10 mV. The elemental distribution in perovskite film was characterized using PHI nanoTOF II Time-of-Flight SIMS.

The conductivity of the films was measured using a diode configuration of glass/ITO/SnO_2_/with or without OI/Au by taking current–voltage curves with a voltage range from −1 to 1 V. The conductivity can be obtained through the following equations:

1 $$V = \frac{I}{{R\left( {{\text{resistance}}} \right)}}$$

2 $$R = p\frac{L}{S}$$

3 $$\sigma = \frac{1}{p} = \frac{VL}{{IS}}$$

where $\sigma$ is the conductivity, *I* is the current density, *L* is the film thickness of the SnO_2_ layer, *V* is the internal voltage in the device, *S* is the cross-sectional area in the device. The current density–voltage (*J–V*) curves were obtained by using a Keithley 4200-SCS source-measure unit.

## Results and Discussion

### Effects of OI Complexes Modification on the Properties of SnO_2_

**Fig. 1:**
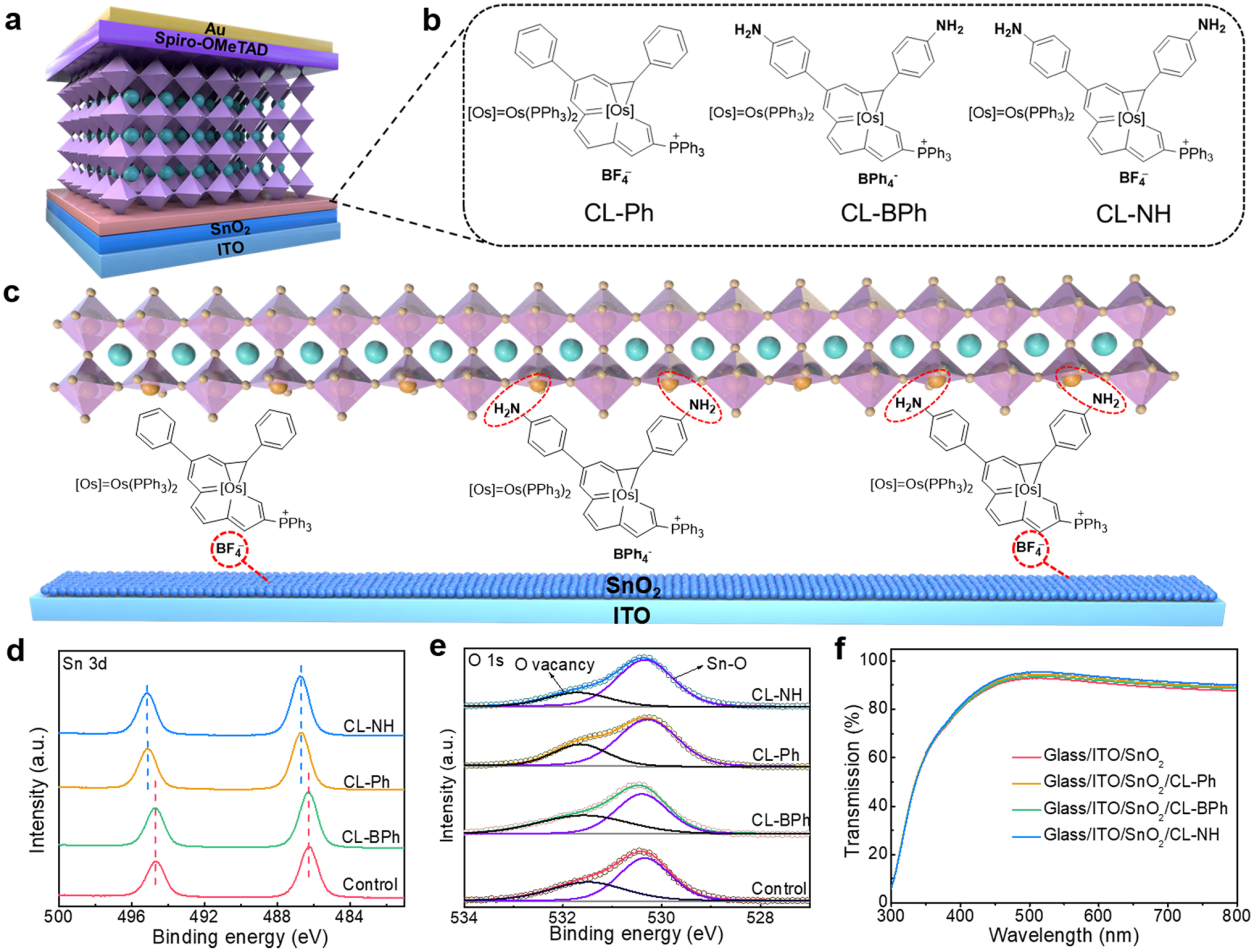
**a** Schematic of the device structure. **b** Chemical structures of CL–Ph, CL–BPh and CL–NH. **c** Schematic diagram of the formation of the passivation layer between the SnO_2_ ETL and perovskite layer. **d** XPS high-resolution spectra of SnO_2_ and SnO_2_/CL–Ph, CL–BPh, CL–NH for Sn 3*d*, and **e** O 1*s*. **f** Optical transmission spectra of SnO_2_, SnO_2_/CL–Ph, SnO_2_/CL–BPh, SnO_2_/CL–NH films on ITO substrates

To achieve multifunctional buried interface layer, a series of OI complexes (CL–Ph, CL–BPh, CL–NH) were rationally designed and synthesized. The corresponding synthetic procedures were detailly described in the Experimental Section. The nuclear magnetic resonance (NMR), high-resolution mass spectra (HRMS) characterizations of CL–Ph, CL–BPh and CL–NH are shown in Figs. S1–S22. Thermogravimetric analysis (TGA, Fig. S23) suggests that the initial decomposition temperatures (T5) measured at the point of 5% weight loss are 242.9, 224.1 and 237.8 °C for CL–Ph, CL–BPh and CL–NH, respectively. The superior thermal properties of OI complexes make it an ideal candidate to serve as buried interface layer with a device structure of indium tin oxide (ITO)/tin oxide (SnO_2_)/OI complexes/perovskite/Spiro-OMeTAD/Au, as shown in Fig. 1a. The chemical structures of the complexes are presented in Fig. 1b, in which it can be seen that CL–Ph complex possess pseudohalide BF_4_^**−**^ anion, CL–BPh complex possess amine (−NH_2_) group, while CL–NH complex contains both BF_4_^**−**^ and −NH_2_ functional groups. Previous studies have demonstrated that halide anions, such as F^**−**^ and Cl^**−**^, can eliminate oxygen vacancies on the SnO_2_ surface, thus promoting device efficiency [16, 26-28]. Accordingly, as shown in Fig. 1c, we assumed that the CL–Ph and CL–NH complexes with pseudohalide BF_4_^−^ anion can interact with SnO_2_ and passivate the electron-poor and -rich defect states of SnO_2_ surface. Besides, the −NH_2_ functional group on CL–BPh and CL–NH tends to interact with Pb^2+^ ions and show positive effect on perovskite crystallization [29]. With the above features, it is believed that the CL–NH complex can significantly enhance the device performance as multifunctional buried interface layer.

To verify our hypothesis, XPS measurements were conducted to investigate the chemical states on the surface of control and OI-SnO_2_ films. Figure 1d presents Sn 3*d* core spectra of the films, in which it can be seen that the Sn peaks of the CL–Ph and CL–NH modified SnO_2_ films shifted to higher binding energy compared with that of the control and CL–BPh SnO_2_ film [30]. This energy shift is mainly ascribed to the interaction between high electron negative F atoms in BF_4_^−^ anion and SnO_2_ films, which is further verified by the lower energy shift of F 1*s* spectra shown in Fig. S24. Besides, O 1*s* core spectra of the films reveal an asymmetric profile (Fig. 1e), which can be deconvoluted into two peaks related to Sn–O bond at 530.32 eV and oxygen vacancies (O_V_) and surface absorbed hydroxyl (O_OH_) at 531.63 eV [31]. By calculating the corresponding peak areas (Table S1), it is found that O_V_ + O_OH_ content in CL–Ph (29.6%) and CL–NH (24.9%) modified SnO_2_ films are much lower than that of control (40.2%) and CL–BPh (45.2%). In view of comparable O_OH_ content of all films due to the same deposition and storage environment, the lower content of O_V_ + O_OH_ in CL–Ph and CL–NH modified SnO_2_ films is mainly attributed to the reduction of O_V_. The results suggest that CL–Ph and CL–NH complexes with BF_4_^−^ anion can strongly interact with SnO_2_ and eliminate O_V_, thus inhibiting the accumulation and recombination of electrons at the surface. In addition, the optical transmission spectra of the films shown in Fig. 1f suggest that OI complexes modification will not deteriorate the transmission of the substrate, ensuring superior visible light utilization of the device. Besides, the conductivity of the tin oxide was determined, and the corresponding current–voltage (*I–V*) curves of the devices based on different tin oxide are shown in Fig. S25. Accordingly, the conductivity of control, CL–BPh, CL–Ph, and CL–NH treated SnO_2_ films are 2.25 × 10^−3^, 2.28 × 10^−3^, 3.04 × 10^−3^ and 3.10 × 10^−3^ S cm^−1^, respectively. The enhancement of conductivity for CL–Ph and CL–NH treated SnO_2_ is mainly attributed to the reduced O_V_ due to the interaction between BF_4_^−^ anion and SnO_2_.

**Fig. 2:**
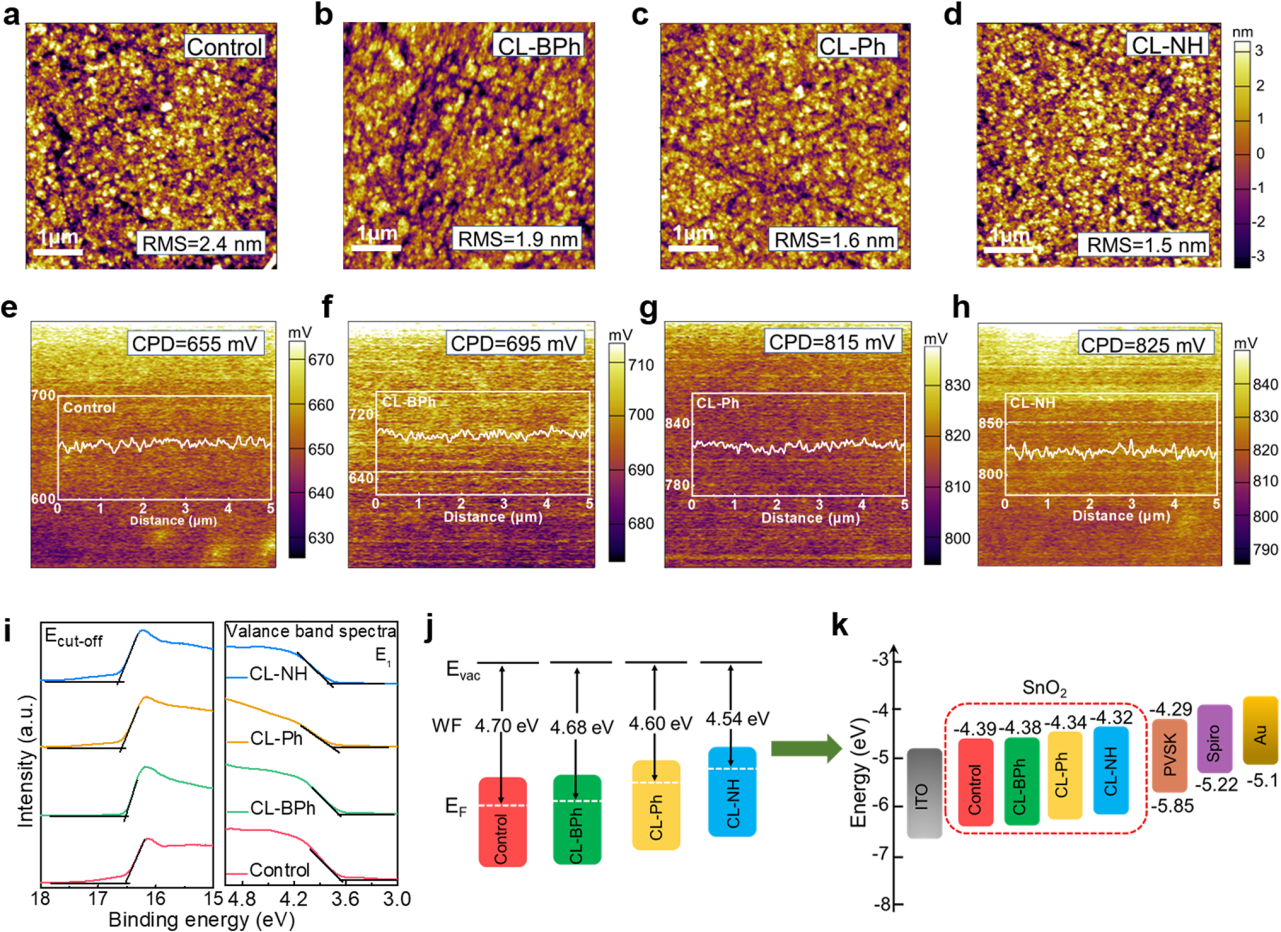
**a–d** AFM topographical images of SnO_2_, SnO_2_/CL–Ph, SnO_2_/CL–BPh, SnO_2_/CL–NH films. **e–h** KPFM of SnO_2_, SnO_2_/CL–Ph, SnO_2_/CL–BPh, SnO_2_/CL–NH films. **i** UPS spectra of secondary electron cutoff and valence bands for SnO_2_, SnO_2_/CL–Ph, SnO_2_/CL–BPh, SnO_2_/CL–NH films, respectively. **j–k** Scheme of energy level alignment

To evaluate the impact of OI complexes modification on the surface morphology of ITO/SnO_2_ films, top-view SEM and atomic force microscopy (AFM) were conducted. The surface SEM images of the films, shown in Fig. S26, suggest that OI complexes modification does not change the morphology of ITO/SnO_2_ film, while the presence of osmium (Os), boron (B) and fluorine (F) elements in the energy-dispersive spectroscopy (EDS) mapping of the corresponding films indicates that OI complexes have been successfully spin-coated on the surface of ITO/SnO_2_ film. Figure 2a–d shows the AFM images of the films, from which we can clearly see that the root mean square (RMS) decreased from 2.4 to 1.9, 1.6, and 1.5 nm for control, CL–BPh, CL–Ph, and CL–NH films, respectively. The smoother surface after OI complexes modification can facilitate charge carrier extraction from perovskite to ETL. Besides, the surface potential of the films was also studied by Kelvin probe force microscopy (KPFM), and the results are presented in Fig. 2e–h. According to the line profile results, the mean contact potential difference (CPD) of the films increased from 655 to 695, 815, and 825 mV for control, CL–BPh, CL–Ph, and CL–NH films, respectively. It is known that the work functions (WFs) of sample can be estimated from the tip work function subtracting the measured CPD value [32]. In this case, the largest CPD value derived from CL–NH film delivers to a smallest work function due to the interaction between BF_4_^−^ anion in CL–NH complex and SnO_2_.

Further, the electronic structures of the SnO_2_ with and without OI complexes modification were investigated using ultraviolet photoelectron spectroscopy (UPS). Figure 2i shows the obtained secondary electron cutoff (E_cutoff_) and valance band spectra of the films. According to the equation: ${\text{E}}_{{\text{F}}} = {\text{E}}_{{{\text{cutoff}}}} - 21.22$ eV, the Fermi level (E_F_) of bare SnO_2_ and CL–BPh, CL–Ph and CL–NH modified SnO_2_ films are calculated to be −4.70, −4.68, −4.60, and −4.54 eV, respectively, as shown in Fig. 2j. Besides, based on the semiconductor band structure, the valance band maximum (*E*_VBM_) of the films can be calculated from the equation: $E_{VBM} = E_{F} + E_{1}$, where E_1_ is the position of VBM to the Fermi level as obtained from the valance band spectra shown in Fig. 2i [7]. Further, the conduction band minimum (*E*_CBM_) of the films can be ascertained through the equation: $E_{CBM} = E_{VBM} + E_{g}$, where *E*_g_ is the optical bandgap derived from the UV–Vis absorption (Fig. S27). The corresponding band structure parameters are summarized in Table S2. Figure 2k presents the energy level diagram of the devices based on different ETL, from which it can be observed that CL–NH modified SnO_2_ film shows a smallest energy gap of 0.03 eV with perovskite layer, thus facilitating photoexcited electrons extraction.

### Influence of OI Complexes on Perovskite Film Crystallinity and Morphology

**Fig. 3:**
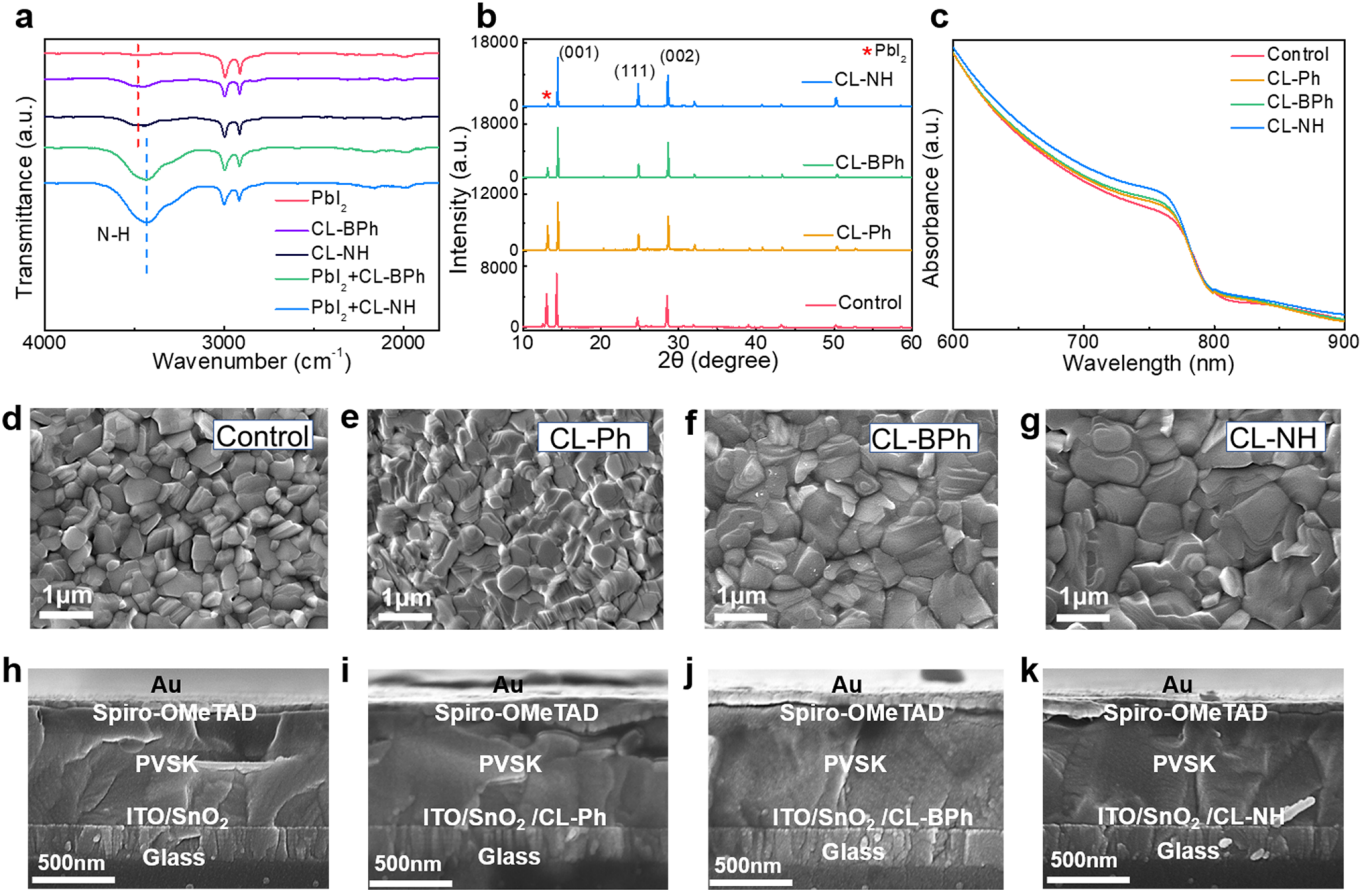
**a** FTIR spectra of PbI_2_, CL–BPh, CL–NH, PbI_2_ + CL–BPh and PbI_2_ + CL–NH. **b** XRD patterns of perovskite films on top of control, CL–Ph-modified SnO_2_, CL–BPh-modified SnO_2_, CL–NH-modified SnO_2_. **c** UV–vis absorption spectra of perovskite films on top of control, CL–Ph-modified SnO_2_, CL–BPh-modified SnO_2_, CL–NH-modified SnO_2_. **d–g** The surface SEM images of perovskite films on top of control CL–Ph-modified SnO_2_, CL–BPh-modified SnO_2_, CL–NH-modified SnO_2_.**h–k** Cross-sectional SEM images of PSC devices based on SnO_2_, CL–Ph, CL–BPh and CL–NH -modified SnO_2_

Moreover, the influence of OI complexes modification on perovskite layer was further validated using a suite of spectroscopy probes. Figure 3 a shows the FTIR spectra of the pure CL–BPh, CL–NH, PbI_2_, PbI_2_ + CL–BPh, and PbI_2_ + CL–NH that dissolved in DMSO. It is noted that the stretching vibration of N–H bond shifted from 3480 cm^−1^ in pure OI complexes (CL–BPh, CL–NH) to a lower wavenumber of 3432 cm^−1^ for the PbI_2_ + CL–BPh and PbI_2_ + CL–NH samples [33]. The lower wavenumber shifted characteristic peak suggests an interaction between −NH_2_ group and PbI_2_, which will affect the perovskite crystallization. To evaluate the crystallinity of the corresponding perovskite films, XRD measurements were conducted, and the results are shown in Fig. 3b. Three characteristic peaks at 14.0°, 24.4°, and 28.2°, which correspond to (001), (111), and (002) crystalline planes of α-phase FAPbI_3_ [34], respectively, can be observed, indicating that OI complexes modification will not affect the crystal structure of the perovskite films. In addition, the strongest (001) peak diffraction intensity coupled with the smallest full width at half maximum (FWHM) value (Fig. S28) suggests that CL–NH film has an improved crystallinity. Besides, it is noted that introduction of OI complexes, especially for −NH_2_ group contained CL–BPh and CL–NH, can significantly reduce the peak intensity of PbI_2_ located at 12.8°. This much reduced residual PbI_2_ is mainly attributed to the porous PbI_2_ film (Fig. S29) due to the interaction between −NH_2_ group and PbI_2_, which can facilitate the penetration of organic salt and enhance the crystallinity of the resulting perovskite film [35]. Moreover, it should be noted that the (111) peak diffraction intensity of the OI-treated perovskite films is much higher than that of control, indicating an enhancement of (111) facet. According to Ma et al. [36], (111) perovskite facet reveals much better hydrophobicity than other facets. In this case, the OI-treated perovskite films perform better water-resistant ability, as supported by the contact angle results shown in Fig. S30. The UV–vis absorption spectra shown in Fig. 3c suggest that CL–NH complex modified film shows the strongest absorption during the visible region, and not affect the bandgap of the perovskite materials (Fig. S31), which is mainly attributed to the best crystallinity as manifested by XRD results [37].

To further explore the effect of OI modification on perovskite film crystallization, surface morphology of the films was studied using SEM, and the results are presented in Fig. 3d–g. It is clear that all films have grown with full coverage of substrates. Compared with control and CL–Ph films, CL–BPh and CL–NH films show larger grain size. Besides, the corresponding AFM images, shown in Fig. S32, suggest that OI complexes modification can reduce the RMS value of the resulting perovskite films. Figure 3h–k shows the cross-sectional SEM images of the devices based on control, CL–Ph, CL–BPh and CL–NH perovskite films. It also can be seen that CL–BPh and CL–NH perovskite film show much larger grain size with less grain boundaries (Fig. S33), which is mainly attributed to the enhanced film crystallinity due to the interaction between −NH_2_ group and PbI_2_. The excellent morphology of the CL–BPh and CL–NH perovskite films is mainly attributed to the interaction between −NH_2_ group and bottom PbI_2_, which contributes to highly crystallized perovskite film, as discussed earlier. The better crystallized perovskite film with large grains and less grain boundaries provides a more favorable environment for charge carrier transportation and extraction, thus enhancing device performance [38].

### Performance of Perovskite Device with OI Complexes Modification

**Fig. 4:**
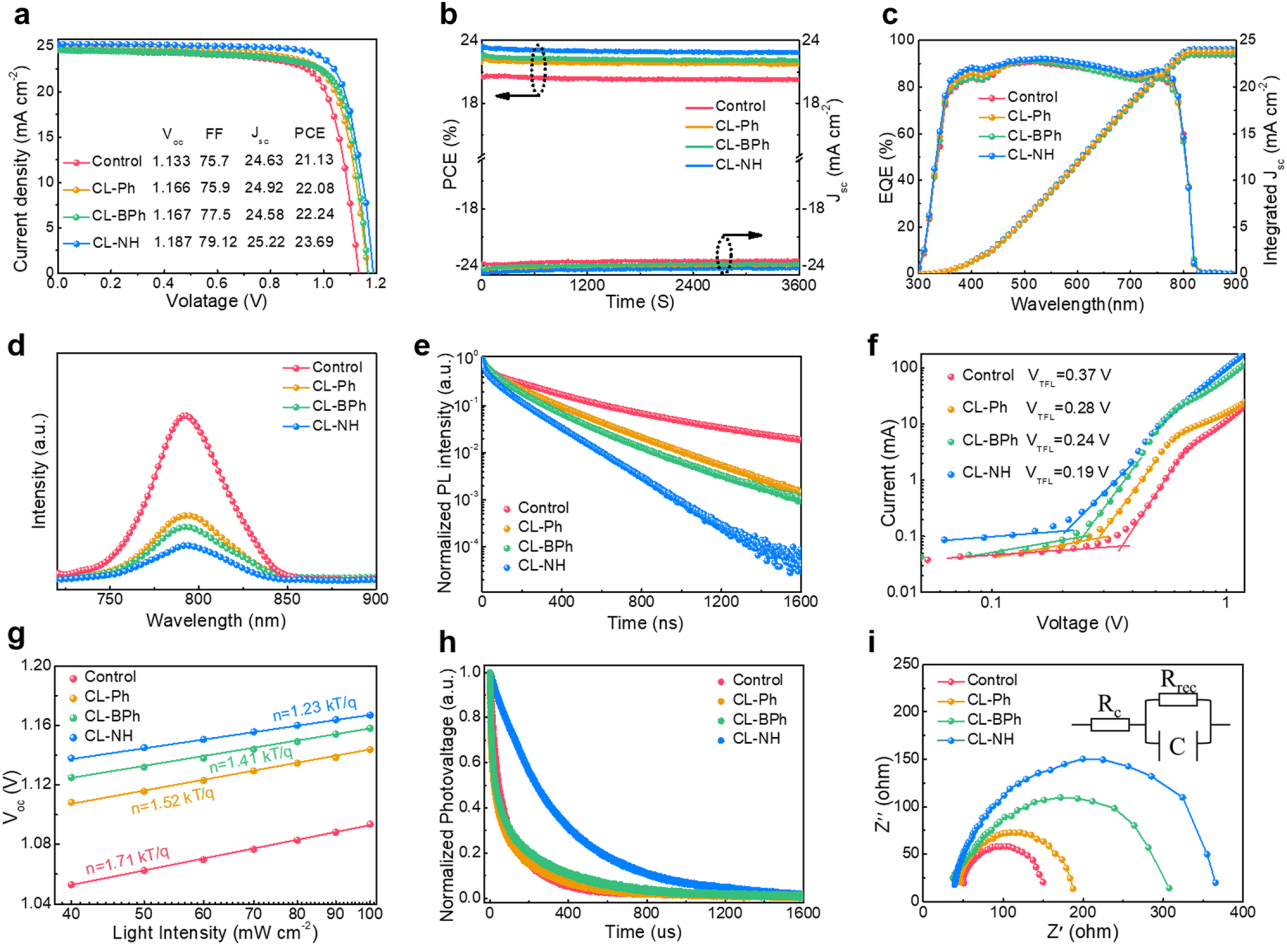
**a** *J-V* curves of CL–Ph-modified SnO_2_, CL–BPh-modified SnO_2_, CL–NH-modified SnO_2_ and control devices. **b** steady-state photocurrent and output PCE at the maximum power point. **c** EQE spectra and the corresponding integrated *J*_sc_. **d** Steady-state PL and **e** TRPL spectra of perovskite films on top of control and CL–Ph-modified SnO_2_, CL–BPh-modified SnO_2_, CL–NH-modified SnO_2_. **f** SCLC plots of electron-only devices (ITO/SnO_2_/perovskite/PCBM/Ag). **g** *V*_oc_ to the light intensity plots of PSCs. **h** TPV decay curves of the devices. **i** Nyquist plots for the different devices

To confirm the effectiveness of OI complexes modification on device performance, PSCs with a planar structure of indium tin oxide (ITO)/SnO_2_/OI complexes/perovskite/spiro-OMeTAD/Au (Fig. 1a) were fabricated. Figure 4a shows the photocurrent density–voltage (*J–V*) curves of the champion devices based on different OI complex modified SnO_2_, and the corresponding photovoltaic parameters are summarized in Table S3. It can be seen that OI complex modification can enhance device efficiency from 21.13% (control) to over 22%. The PCE enhancement for devices based on CL–Ph film can be ascribed to the reduction of O_V_ and better aligned energy level at the ETL/perovskite interface due to the interaction between BF_4_^−^ anion and SnO_2_, while the efficiency enhancement for CL–BPh film-based devices is mainly derived from high-quality perovskite film as a result of the introduction of −NH_2_ group. Interestingly, with both BF_4_^−^ anion and −NH_2_ functional group, CL–NH complex modified SnO_2_ contributes to devices with a highest PCE of 23.69%. To verify the reliability of the *J–V* curves, steady-state output (SPO) of the devices at the maximum power point (MPP) was tracked for 3600 s, as shown in Fig. 4b. The PCE of the devices based on control, CL–Ph, CL–BPh and CL–NH film stabilized at 20.27%, 21.75%, 22.10%, and 22.90%, respectively, which are consistent with the values obtained from *J–V* measurements. Figure 4c presents the external quantum efficiency (EQE) spectra of the devices, from which the integrated *J*_sc_ can be calculated to be 23.42, 23.48, 23.81, and 24.08 mA cm^−2^ for control, CL–Ph, CL–BPh, and CL–NH device, respectively, matching well with the *J–V* curves. The statistical PCEs derived from 30 independent PSCs are presented in Fig. S34 from which it can be seen that devices based on different perovskite films exhibit high reproducibility.

To validate the efficiency enhancement, charge carrier kinetics of the devices were systematically studied. Figure 4d shows the steady-state PL spectra of the perovskite films fabricated on ETL. It is noted that after OI complexes modification, the perovskite film with a faster PL quenching, indicating a more efficient charge carrier extraction process, which is beneficial for device performance. Time-resolved PL (TRPL) was further conducted to determine the charge carrier lifetime of the films [39], and the results were fitted by a bi-exponential decay function with detail parameters summarized in Table S4. As shown in Fig. 4e, the average carrier lifetime of the control, CL–Ph, CL–BPh, and CL–NH film are 271.5, 207.9, 168.8, and 124.4 ns, respectively. The shortest carrier lifetime in the CL–NH film agrees well with its fastest PL quench, which are mainly derived from the reduced O_V_ and better aligned energy level as discussed earlier, this means that the charge transfer efficiency is enhanced [40, 41]. Besides, space-charge-limited current (SCLC) technique was adopted to quantitatively evaluate the defect density of perovskite films. Figure 4f shows the dark *J–V* curves of the electron-only devices with a structure of ITO/SnO_2_/OI complexes/Perovskite/PCBM/Ag. It can be seen that the *V*_TFL_ of control, CL–Ph, CL–BPh, and CL–NH devices are 0.37, 0.28, 0.24, and 0.19 V, respectively. According to the equation: $N_{t} = \frac{{2V_{TFL} \varepsilon_{r} \varepsilon_{0} }}{{eL^{2} }}$, where ε_0_ is the vacuum permittivity, ε_r_ is the relative dielectric constant perovskite (ε_r_ = 62.23), e is the electron charge [42, 43], and L is the thickness of the film, which is ≈500 nm according to Fig. 3h, the trap densities of the perovskite films are calculated to be 1.02 × 10^16^ (control), 7.71 × 10^15^ (CL–Ph), 6.61 × 10^15^ (CL–BPh), and 5.23 × 10^15^ (CL–NH) cm^−3^, respectively. The lowest trap density for CL–NH film is mainly attributed to its superior crystallinity. Then, light intensity dependent *J*_sc_ and *V*_oc_ measurements were also conducted to investigate charge extraction and recombination of the devices. The *J*_sc_ versus light intensity of the devices reveal a linear relationship (Fig. S35), indicating a favorable environment for charge extraction. While, the most ideal α value of 0.96 for CL–NH device suggests the formation of high-quality perovskite film with better aligned energy level at ETL/perovskite interface, thus facilitating charge extraction and collection. Figure 4g shows the light dependent *V*_oc_ results of the devices, in which it can be seen that OI devices exhibit a reduction of slope. It has been reported that the deviation of the slope from unity kT q^−1^ indicates the trap-assisted recombination in PSCs. In this case, the smallest slope of 1.23 kT q^−1^ suggests that the trap-assisted recombination within the CL–NH device was substantially suppressed, which results in a significant enhancement of *V*_oc_ [44, 45]. In addition, transient photovoltage (TPV) and transient photocurrent (TPC) measurements were conducted to better realize the charge recombination and extraction kinetics. As presented in Fig. 4h, the TPV results reveal that compared with decay time of 152.3 μs of the control device, CL–NH device demonstrates a slowest decay time of 352.4 μs, suggesting that the charge recombination within CL–NH device is effectively suppressed. While, according to the TPC results shown in Fig. S36, the fastest charge extraction time of 0.84 μs derived from CL–NH device indicates an enhanced charge extraction efficiency, which is responsible for the highest *J*_sc_ [46, 47]. Further, Nyquist plots shown in Fig. 4i reveal that CL–NH device has a largest semicircle, which corresponds to a largest recombination resistance (*R*_rec_), suggesting that the charge recombination process is effectively suppressed within the device [48, 49]. Overall, the efficient charge extraction, suppressed charge recombination, and reduced trap density are seen as the main reasons for the significant efficiency enhancement in the CL–NH device.

### Effect of OI Complexes Modification on Device Stability

**Fig. 5:**
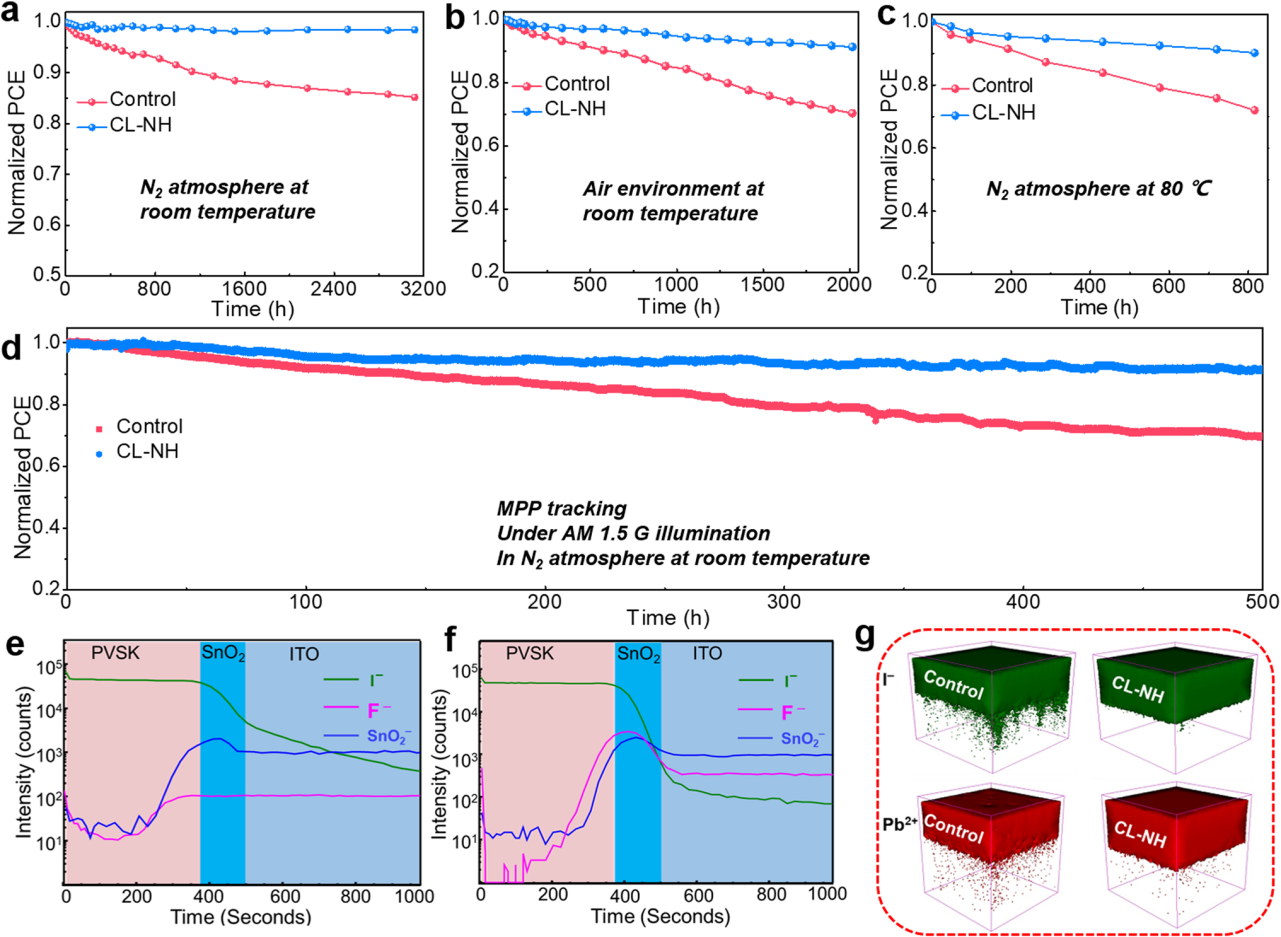
**a** Stability measurements of unencapsulated devices based on different films in a glovebox at room temperature, **b** ambient environment with RH of 20–30%, **c** glovebox at 85 °C. **d** Maximum power point (MPP) tracking with and without internal encapsulation in N_2_ atmosphere under 100 mW cm^−2^ white light illumination. ToF–SIMS depth profiles with the structure **e** SnO_2_/perovskite and **f** SnO_2_/CL–NH/perovskite. **g** The corresponding ion distribution of I^−^ and Pb^2+^ in perovskite film

In addition to device efficiency, stability is another important indicator for evaluating device performance [50, 51]. Therefore, the long-term, air and thermal stability of the unencapsulated devices were measured and compared systematically. Figure 5a shows the long-term stability of the devices stored in glove box with inert atmosphere at room temperature. It is noted that after CL–NH modification, the resulting device exhibits superior stability with negligible PCE loss after more than 3000 h, while control device present continuous attenuation. Ambient stability of the devices under air environment with humidity of 20%–30% was further studied to evaluate practical application potential [52, 53]. As shown in Fig. 5b, after 2000 h storage, CL–NH device maintains over 91% of its initial PCE, while the control device only retains 65% of its original value. Besides, stability results under nitrogen atmosphere at 80 °C for 800 h suggest that CL–NH device performs much better thermal stability than that of control, which was attributed to the suppressed of ion migration of perovskite (Fig. 5c) [54-57]. To further evaluate the operational lifetime of the unencapsulated devices in a real-world, maximum power point (MPP) tracking under 100 mW cm^−2^ white light illumination was conducted [58, 59]. Figure 5d presents the MPP results of the devices, from which it can be observed that CL–NH device maintains 91.5% of its original efficiency after 500 h tracking. While the control device suffered a severe PCE degradation with only 69% of its initial PCE value retained. In general, ions migration in PSCs is detrimental to device performance and stability, and lower defect densities provide fewer channels for ion migration. CL–NH complex with −NH_2_ functional group can strongly interact with PbI_2_ and enhance the perovskite crystallinity, contributing to perovskite film with lower defect densities, thus inhibiting ion migration.

To better realize the suppression of ion migration, we further conducted time-of-flight secondary ion mass spectrometry (ToF–SIMS), as shown in Fig. 5e–g. The results suggest that the control perovskite shows a severe I^−^ diffusion with a high content in the bottom SnO_2_ and ITO (Fig. 5e). While, after the introduction of CL–NH, the migration of I^−^ is effectively suppressed with a very low content in SnO_2_/ITO (Fig. 5f). Besides, the corresponding 3D image for I^−^ and Pb^2+^ distribution shown in Fig. 5g further validate the inhibited I^−^ and Pb^2+^ migration, which is mainly derived from the improved structural stability of PbI_6_ octahedral frameworks due to the interaction between −NH_2_ functional groups and Pb^2+^ [60]. These results demonstrate convincingly that CL–NH modification can effectively enhance the overall stability of the devices, which can be attributed to three points. First, the highly crystallized perovskite film with large grains and preferred (111) facet can reduce the pathways for moisture ingress and enhance the hydrophobicity, thus suppressing film degradation. Second, the interaction between −NH_2_ group and perovskite can inhibit ion migration during heating, thereby enhancing the thermal stability. Third, the excellent energy level alignment at the ETL/perovskite interface can effectively play the roles in dual-passivation of interfacial defects, and the strong interaction between CL–NH and perovskite can suppress SnO_2_/perovskite interface ion migration, which is essential for the long-term stability of the devices, thereby contributing to the enhanced stability of PSCs significantly.

## Conclusions

In summary, we have designed and synthesized a series of OI complexes as multifunctional interface materials to achieve high efficiency two-step method-based PSCs. The results suggest that the optimal CL–NH complex with BF_4_^−^ functional group can not only passivate oxygen vacancies on the surface of SnO_2_ but also balance the energy level between SnO_2_ and perovskite, thus inhibiting the accumulation and recombination of electrons at the interface. Besides, it is demonstrated that −NH_2_ group in CL–NH complex can strongly interact with PbI_2_ and modulate the crystallization of perovskite, resulting in highly crystallized film with large grains and less grain boundaries. As a result, the fabricated device with CL–NH modification achieves a PCE of 23.69%, which is much higher than that of control (21.13%). Furthermore, the resulting unencapsulated device performs excellent light soaking stability with 91.5% initial PCE retained after 500 h MPP tracking under continuous 100 mW cm^−2^ light illumination in N_2_ atmosphere. Our results provide insights on interface modification and a new avenue to improve the quality of the buried interface at the regular PSCs for pursuing efficient and stable perovskite photovoltaic devices.

### Supplementary Information

Below is the link to the electronic supplementary material.

Supplementary file1 (PDF 2986 KB)
