# Supplementary material for: Synergistic Optimization of Buried Interface by Multifunctional Organic–Inorganic Complexes for Highly Efficient Planar Perovskite Solar Cells
Source: Nanomicro Lett. 2023 Jun 19;15:156. doi: 10.1007/s40820-023-01130-5 (PMC10279600; doi:10.1007/s40820-023-01130-5)
Supplement: Supplementary file 1 — Supplementary file1 (PDF 2986 KB) [file 40820_2023_1130_MOESM1_ESM.pdf]

Supporting Information for

Synergistic Optimization of Buried Interface by Multifunctional Organic-Inorganic Complexes for Highly Efficient Planar Perovskite Solar Cells

Heng Liu[1, 2, #], Zhengyu Lu[3, #], Weihai Zhang[2, #], Hongkang Zhou[2], Yu Xia[2], Yueqing Shi[4], Junwei Wang[2], Rui Chen[4], Haiping Xia[3, *] and Hsing-Lin Wang[2, 5, *]

[1]School of Materials Science and Engineering, Harbin Institute of Technology, Harbin 150001, P. R. China

[2]Department of Materials Science and Engineering, Southern University of Science and Technology, Shenzhen 518055, Guangdong, P. R. China

[3]Shenzhen Grubbs Institute and Department of Chemistry, Southern University of Science and Technology, Shenzhen 518055, Guangdong, P. R. China

[4]Department of Electrical and Electronic Engineering, Southern University of Science and Technology, Shenzhen 518055, Guangdong, P. R. China

[5]Key University Laboratory of Highly Efficient Utilization of Solar Energy and Sustainable Development of Guangdong, Southern University of Science and Technology, Shenzhen 518055, Guangdong, P. R. China

[#]Heng Liu, Zhengyu Lu and Weihai Zhang contributed equally to this work.

*Corresponding authors. E-mail:wangxl3@sustech.edu.cn (Hsing-Lin Wang); xiahp@sustech.edu.cn (Haiping Xia)

S1 Experimental Section

Materials: ITO glass were purchased from Advanced Election Technology (China). SnO_2 were purchased from Alfa Aesar. Formamidinium Iodide (FAI), Methylammonium Bromide (MABr), Methylammonium Chloride (MACl), 4-tert-butylpyridine and Lithium-bis (trifluoromethanesulfonyl) imide (Li-TFSI) were purchased from Advanced Election Technology (China). Spiro-OMeTAD, Lead (II) iodide PbI_2 were purchased from Xi'an Polymer Light Technology Corp (Xi'an p-OLED). *N*,*N*-dimethylformamide (DMF), dimethylsulfoxide (DMSO), chlorobenzene (CB), isopropyl alcohol (IPA) ,acetonitrile (ACN) and ethanol purchased from Sigma-Aldrich. Gold (Au, 99.99%) were obtained from commercial sources.

Solution Preparation: SnO_2 colloid solution (15 wt %) was diluted within DI water (1:5.5, v: v), and stirring at room temperature for 10min, followed use a syringe and an aqueous filter to filter the above solution. The preparation of PbI_2 precursor solution, 599.3 mg PbI_2 powder was dissolved in 1mL DMF/DMSO 950:50 and stirred overnight at 70 °C. The preparation of the solution of organic amine salts, an isopropyl alcohol (IPA) solution containing organic salts (the mass ratio of FAI: MABr: MACl is 60 mg:6 mg: 6 mg), and stirred at 70 °C for 30 min. The preparation of the solution of Spiro-OMeTAD HTL, which consisted of 72.3 mg spiro-OMeTAD, 28.8 μL 4-tertbutylpyridine, 17.5 μL lithium-bis (trifluoromethanesulfonyl) imide (Li-TFSI) solution (520 mg Li-TFSI in 1 mL acetonitrile), and 1 mL chlorobenzene. The preparation of organic-inorganic (OI) complexes solution, 1 mg CL-BPh, CL-Ph and CL-NH powder was dissolved in 1mL DMF and stirred at room temperature.

Device Fabrication: The glass/ITO substrate was first scrubbed in detergent, and then sequentially cleaned by sonication in deionized water, acetone, and isopropanol for 25 minutes, respectively. After that, nitrogen dried glass/ITO were treated with plasma for 5 min before usage. Then deposit SnO_2 on the substrate as an electron transport layer by spin-coated at 3500 rpm for 30 s and annealed in ambient air at 150 °C for 30 min on the hot plate. After cooling to room temperature, the substrates were transferred to a nitrogen filled glove box. Then, the dissolved CL-BPh, CL-Ph and CL-NH are spin-coated on the surface of the perovskite at 4000 rpm for 30 s. After that the 1.3M of PbI_2 dissolved in anhydrous DMF: DMSO 95:5(v:v) was spin coated onto SnO_2 at 2000 rpm for 30 s, and heating at 70 °C for 1 min and cooling 10 min, followed the mixture solution of FAI: MABr: MACl (60 mg :6 mg :6 mg in 1 mL IPA) was spin-coating onto the PbI_2 at 3000 rpm for 30 s, then transferred into ambient air (RH30%-40%) filled glove box annealed at 150 °C for 10 min on the hot plate. After the substrates were transferred to a nitrogen filled glove box, cooling to room temperature. Subsequently, the substrate transferred into nitrogen filled glove box was deposited on the perovskite films as the hole transport layer by spin-casting the Spiro-OMeTAD solution at 4000 rpm for 30 s. Finally, an approximate 80 nm thick of Au electrode was fabricated using a shadow mask under high vacuum by thermal evaporation.

Characterizations: The crystal structure and phase of the perovskite were characterized using X-ray diffraction spectrometer were obtained on Bruker Advanced D8 X-ray diffractometer using Cu Kα (λ = 0.154 nm) radiation. A UV-Vis spectrophotometer (Agilent Cary 5000) was used to collect the absorbance spectra of the perovskite films. Steady state photoluminescence (PL) spectra were recorded on Shimadzu RF-5301pc. Time-resolved photoluminescence spectra were measured on a PL system (Fluo-Time 300) under excitation with a picosecond pulsed diode laser with a repetition frequency of 1 MHz. The morphology of the films was studied by field-emission scanning electron microscopy (SEM; TESCAM MIRA3). The surface potential of perovskite films obtained with a atomic force microscope (AFM; Asylum Research MFP-3D-Stand Alone). X-ray photoelectron spectroscopy (XPS) was conducted on a Thermo ScientificTM K-AlphaTM+ spectrometer equipped with a monochromatic Al Kα X-ray source (1486.6 eV) operating at 100 W. Samples were analyzed under vacuum ($P < 10^{-8}$ mbar) with a pass energy of 150 eV (survey scans) or 50eV (high-resolution scans). The XPS spectra were calibrated by the binding energy of 284.8 eV for C 1s. Ultraviolet photoelectron spectroscopy (UPS, ESCALAB 250Xi, Thermo Fisher) measurements were carried out using a He Iα photon source (21.22 eV). The current density–voltage (J–V) curves of fabricated devices were obtained from the forward and reverse scan with 30 mV intervals and 10 ms delay time under AM 1.5 G illumination (100 mW cm^{-2}) were collected using a source meter (Keysight B2901A) and a solar simulator (Enlitech SS-F5-3A). The EQE spectra was measured using an quantum efficiency measurement system (Enlitech QER-3011) in which the light intensity at every wavelength was calibrated with a Si detector before measurement. The maximum-power point (MPP) output was measured by testing the steady-state current density at the maximum-power-point voltage. Electrochemical impedance spectroscopy (EIs) was tested with the frequency range from 100 Hz to 1 MHz by the electrochemical workstation (Princeton Applied Research, P4000+) in the dark conditions at with a bias of 1 V. The amplitude is 10 mV. The elemental distribution in perovskite film was characterized using PHI nanoTOF II Time-of-Flight SIMS.

S2 Preparation and Characterization of Organic-inorganic (OI) Complexes

S2.1 General Information

All syntheses were carried out under an inert atmosphere (nitrogen) using standard Schlenk techniques unless otherwise stated. The osmapentalene derivates **1**, **2(CL-Ph)**, **3(CL-NH)**, **4(CL-BPh)** were synthesized according to the published literatures [S1-S3]. The other reagents and solvents were used as purchased from commercial sources without further purification.

Column chromatography was performed on silica gel (200–300 mesh) in air. NMR spectra was collected on a Brucker AVANCE NEO 400 spectrometer (400 MHz) or Brucker AVANCE NEO 600 spectrometer (600 MHz). ^{1}H and ^{13}C{^{1}H} NMR chemical shifts (δ) are relative to tetramethyl silane, and ^{31}P{^{1}H} NMR chemical shifts are relative to 85% H_3PO_4. The absolute values of the coupling constants are given in hertz (Hz). Multiplicities are abbreviated as s (singlet), d (doublet), t (triplet), q (quartet), m (multiplet) and br (broad). The high-resolution mass spectra (HRMS) experiments were performed on a Thermo Scientific Q Exactive instrument.

S2.2 Preparation and Characterization of 2

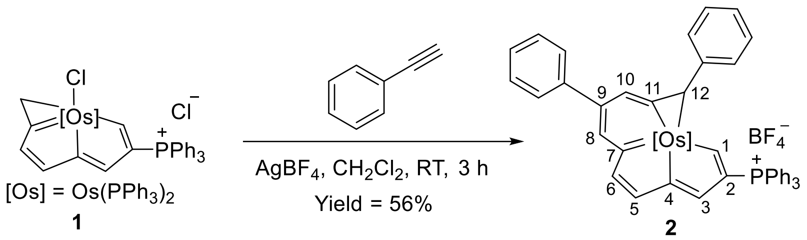

Phenylacetylene (55 µL, 0.50 mmol) was added to a mixture of complex **1** (115 mg, 0.10 mmol) and $AgBF_4$ (58 mg, 0.30 mmol) in 10 mL wet dichloromethane. The reaction mixture was stirred at room temperature for 3 h to give a yellow-green solution, and then the solid suspension was removed by filtration. The volume of the filtrate was reduced under vacuum to approximately 2 mL, and then loaded on silica gel column eluted by dichloromethane/methanol (20/1). The green band was collected, and the solvent was evaporated to dryness under vacuum. The resultant residue was washed with diethyl ether and then dried under vacuum to obtain a green solid of complex **CL-Ph**. Yield, 77 mg, 56%.

^{1}H NMR plus ^{1}H-^{13}C HSQC (400.2 MHz, CD_2Cl_2): δ = 13.12 (d, J_{HH} = 21.3 Hz, 1H, H1), 8.61 (m, 1H, H3), 8.07 (s, 1H, H8), 7.68 (s, 1H, H10), 7.52 (s, 1H, H5), 7.44 (s, 1H, H6), 6.62 (dd, J_{PH} = 13.9 Hz, J_{HH} = 5.3 Hz, 1H, H12), 8.05–5.70 ppm (55H, other aromatic protons). ^{31}P NMR (162.0 MHz, CD_2Cl_2): δ = 9.19 (t, J_{PP} = 7.0 Hz, C*P*Ph3), -8.30 (dd, J_{PP} = 255.1 Hz, J_{PP} = 7.0 Hz, Os*P*Ph3), -19.39 (dd, J_{PP} = 255.1 Hz, J_{PP} = 7.0 Hz, Os*P*Ph3) ppm. ^{13}C NMR plus DEPT-135, ^{1}H-^{13}C HSQC and ^{1}H-^{13}C HMBC (100.6 MHz, CD_2Cl_2): δ = 232.1 (t, J_{PC} = 8.8 Hz, C7), 218.7 (t, J_{PC} = 5.0 Hz, C11), 208.1 (t, J_{PC} = 26.0 Hz, J_{PC} = 5.6 Hz, C1), 200.7 (dt, J_{PC} = 27.0 Hz, J_{PC} = 6.0 Hz, C4), 163.4 (s, C6), 159.2 (s,C5), 159.1 (s,C9), 136.5 (d, J_{PC} = 25.5 Hz, C3), 128.4 (s, C8), 120.7 (d, J_{PC} = 86.9 Hz, C2), 114.4 (s, C10), 14.9 (s, C12), 148.4–124.6 ppm (other aromatic carbons). ^{11}B NMR (128.4 MHz, CD_2Cl_2): δ = -1.09 (s, *B*F4) ppm. ^{19}F NMR (376.5 MHz, CD_2Cl_2): δ = -153.26 (s, B*F*4) ppm. HRMS (ESI): *m/z* calcd for $[C_{78}H_{62}OsP_3]^+$, 1283.3674; found, 1283.3676.

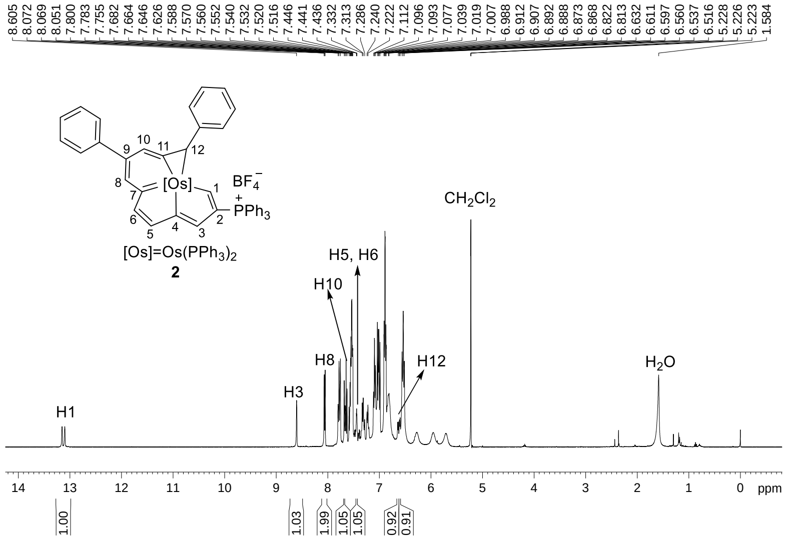

Fig. S1 The ^{1}H NMR (400.2 MHz, CD_2Cl_2) spectrum for complex **CL-Ph**

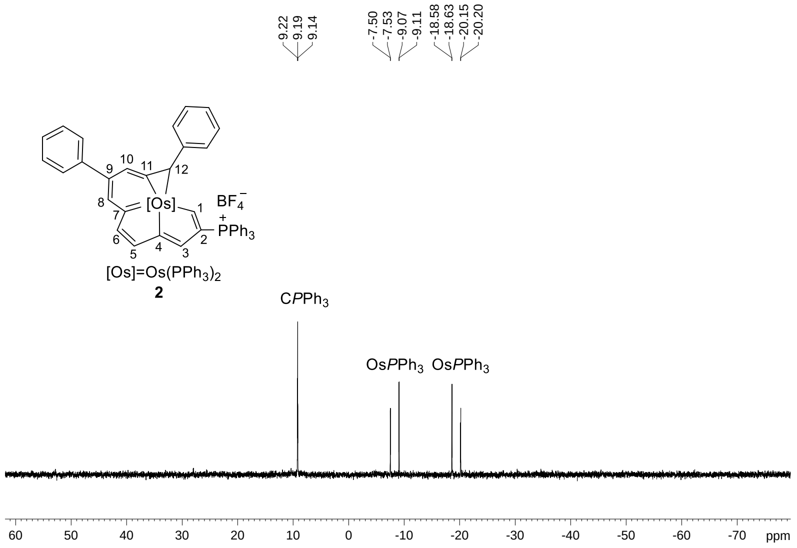

Fig. S2 The ^{31}P{^{1}H} NMR (162.0 MHz, CD_2Cl_2) spectrum for complex **CL-Ph**

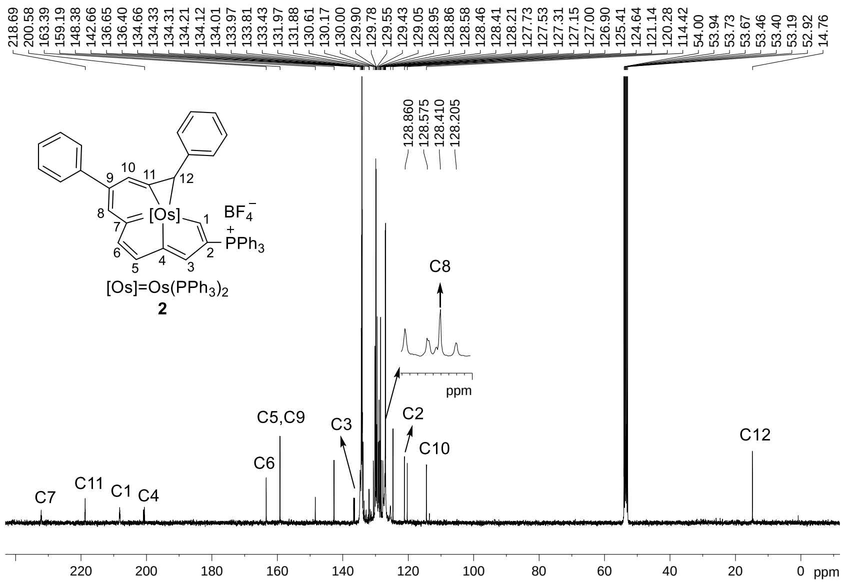

Fig. S3 The ^{13}C{^{1}H} NMR (100.6 MHz, CD_2Cl_2) spectrum for complex **CL-Ph**

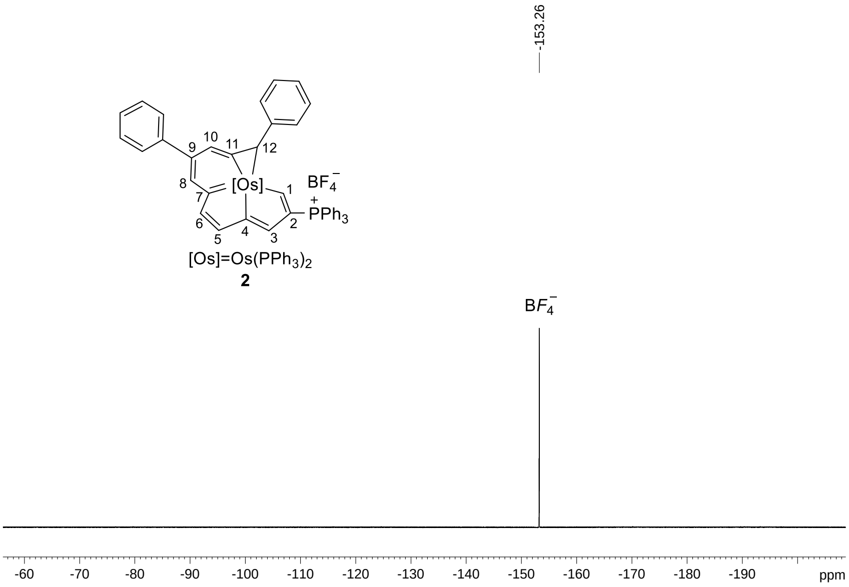

Fig. S4 The ^{11}B{^{1}H} NMR (128.4 MHz, CD_2Cl_2) spectrum for complex **CL-Ph**

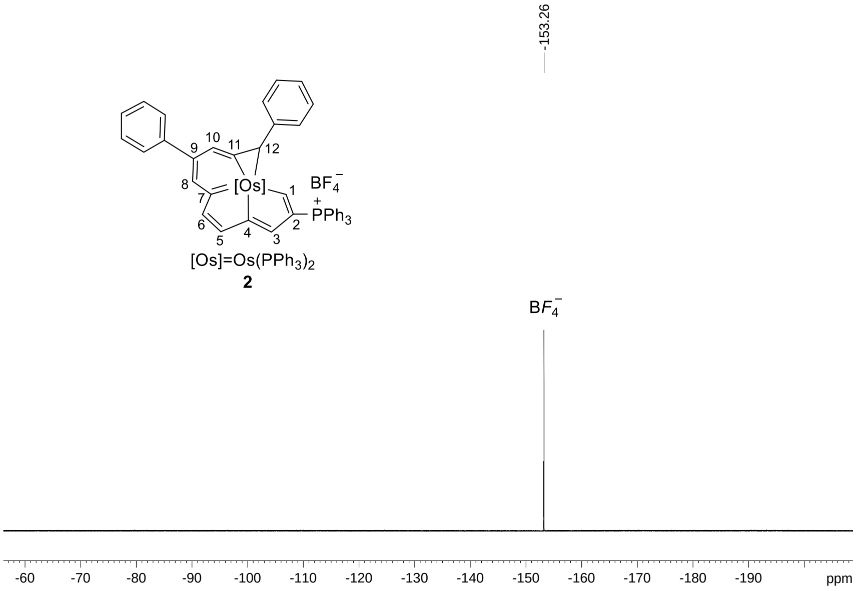

Fig. S5 The ^{19}F{^{1}H} NMR (376.5 MHz, CD_2Cl_2) spectrum for complex **CL-Ph**

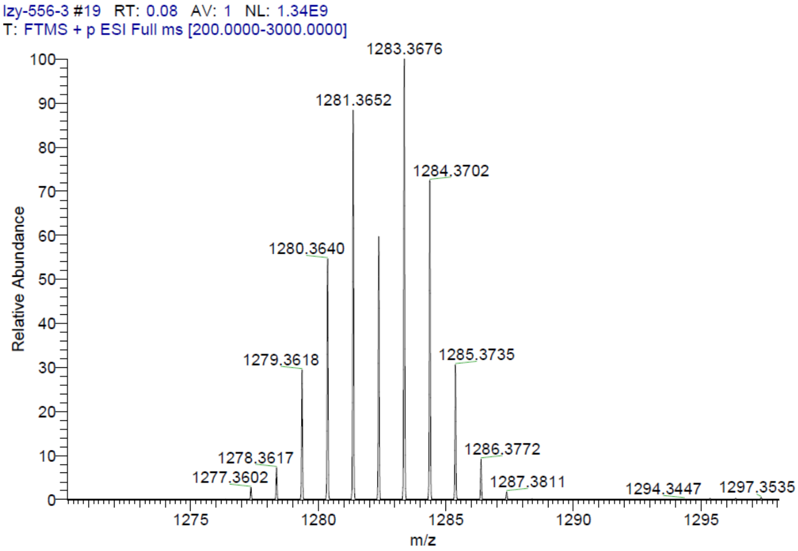

Fig. S6 Positive-ion ESI-MS spectrum of [**CL-Ph**]$^+$ measured in methanol

S2.3 Preparation and Characterization of 3a

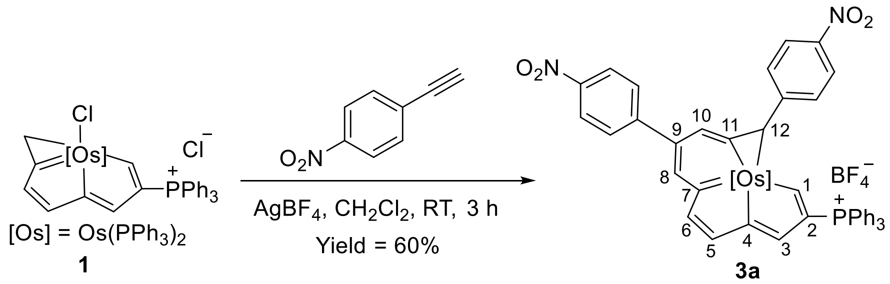

Wet dichloromethane (10 mL) was added to a mixture of complex **1** (115 mg, 0.10 mmol), 1-ethynyl-4-nitrobenzene (74 mg, 0.50 mmol) and $AgBF_4$ (58 mg, 0.30 mmol). The reaction mixture was stirred at room temperature for 3 h to give a yellow-green solution, and then the solid suspension was removed by filtration. The volume of the filtrate was reduced under vacuum to approximately 2 mL, and then loaded on silica gel column eluted by dichloromethane/methanol (20/1). The green band was collected, and the solvent was evaporated to dryness under vacuum. The resultant residue was washed with diethyl ether to obtain a yellow-green solid of complex **3a**, which was dried under vacuum. Yield, 88 mg, 60%.

^1H NMR plus ^1H-^{13}C HSQC (600.1 MHz, CD_2Cl_2): δ = 13.29 (d, J_{HH} = 21.0 Hz, 1H, H1), 8.70 (m, 1H, H3), 7.75 (d, J_{HH} = 4.1 Hz, 1H, H5), 7.71 (s, 1H, H8), 7.70 (s, 1H, H10), 7.66 (m, 1H, H6), 6.93 (m, 1H, H12), 8.48–5.64 ppm (53H, other aromatic protons). ^{31}P NMR (242.9 MHz, CD_2Cl_2): δ = 10.45 (t, J_{PP} = 5.9 Hz, C*P*Ph$_3$), -10.91 (dd, J_{PP} = 252.2 Hz, J_{PP} = 5.9 Hz, Os*P*Ph$_3$), -17.81 (dd, J_{PP} = 252.2 Hz, J_{PP} = 5.9 Hz, Os*P*Ph$_3$) ppm. ^{13}C NMR plus DEPT-135, ^1H-^{13}C HSQC and ^1H-^{13}C HMBC (150.9 MHz, CD_2Cl_2): δ = 229.9 (br, C7), 215.4 (br, C11), 207.3 (m, C1), 201.1 (d, J_{PC} = 24.8 Hz, C4), 164.6 (s, C6), 160.7 (s,C5), 158.4 (s,C9), 137.9 (d, J_{PC} = 25.1 Hz, C3), 129.1 (s, C8), 120.2 (d, J_{PC} = 86.9 Hz, C2), 113.7 (s, C10), 15.3 (s, C12), 154.9–124.0 ppm (other aromatic carbons). ^{11}B NMR (192.6 MHz, CD_2Cl_2): δ = -1.10 (s, *B*F$_4$) ppm. ^{19}F NMR (564.7 MHz, CD_2Cl_2): δ = -153.21 (s, BF_4) ppm. HRMS (ESI): *m/z* calcd for $[C_{78}H_{60}N_2O_4OsP_3]^+$, 1373.3375; found, 1373.3375.

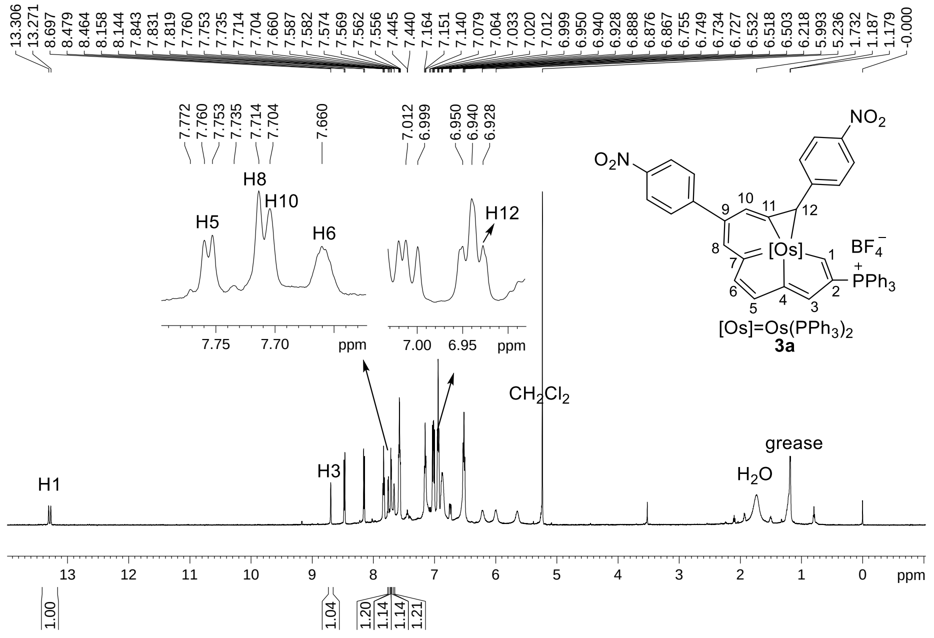

Fig. S7 The ^1H NMR (600.1 MHz, CD_2Cl_2) spectrum for complex **3a**

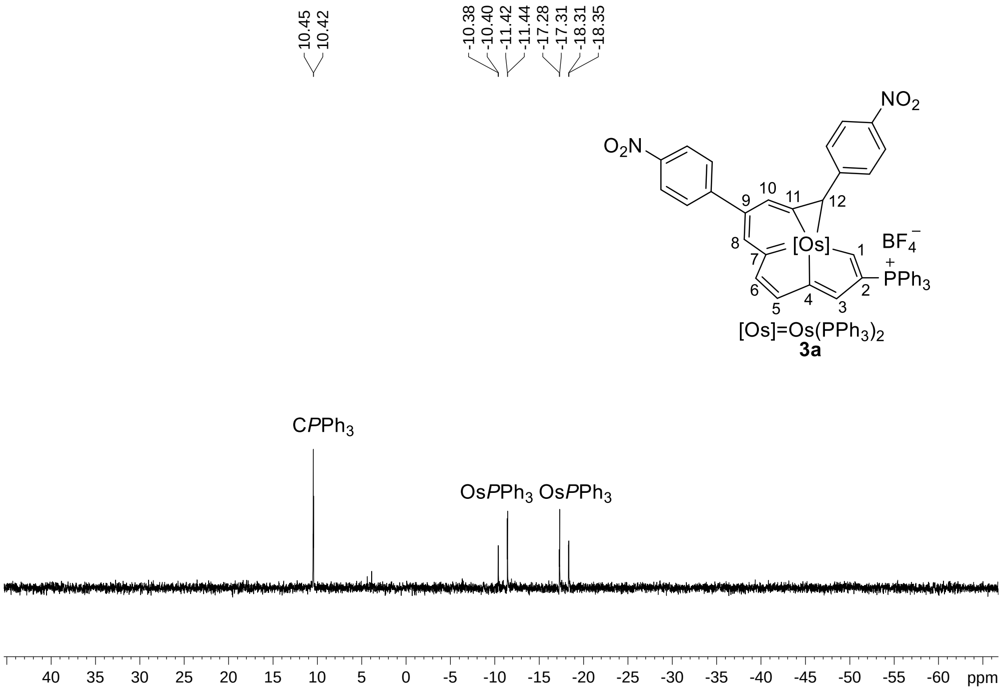

Fig. S8 The $^{31}P\{^{1}H\}$ NMR (242.9 MHz, CD_2Cl_2) spectrum for complex **3a**

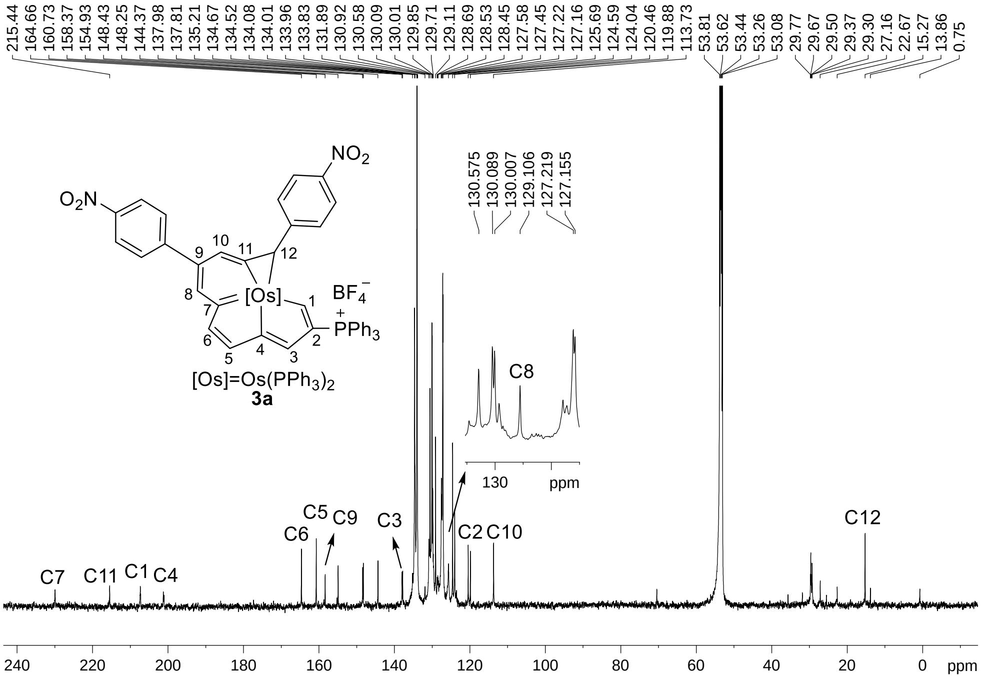

Fig. S9 The $^{13}C\{^{1}H\}$ NMR (150.9 MHz, CD_2Cl_2) spectrum for complex **3a**

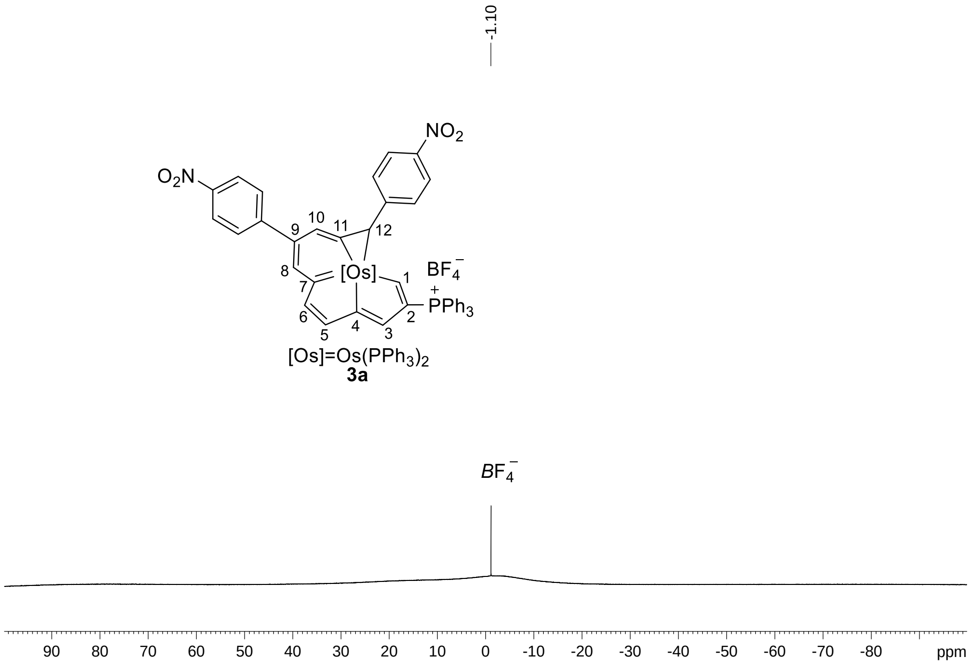

Fig. S10 The ^{11}B{^{1}H} NMR (192.6 MHz, CD_2Cl_2) spectrum for complex **3a**

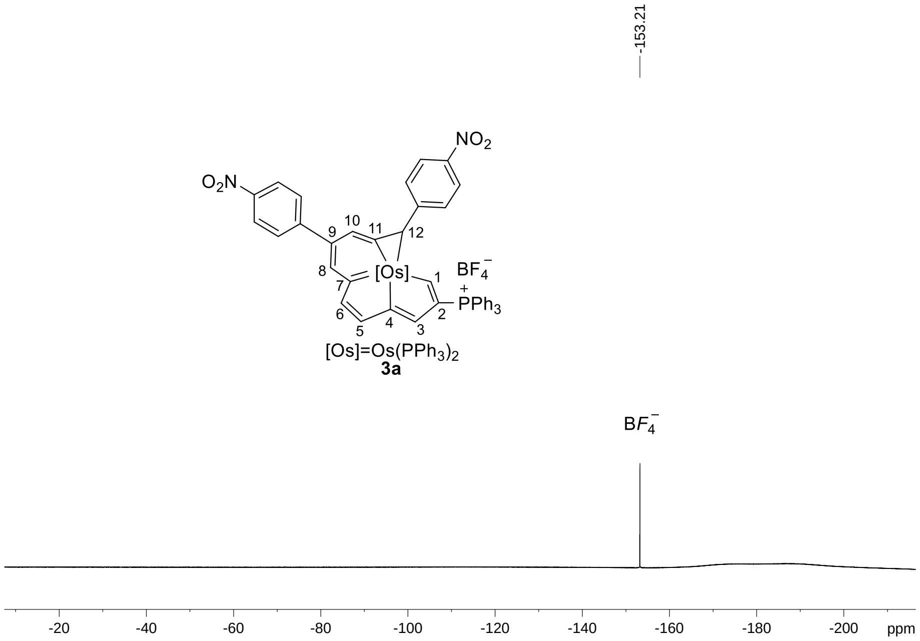

Fig. S11 The ^{19}F{^{1}H} NMR (564.7 MHz, CD_2Cl_2) spectrum for complex **3a**

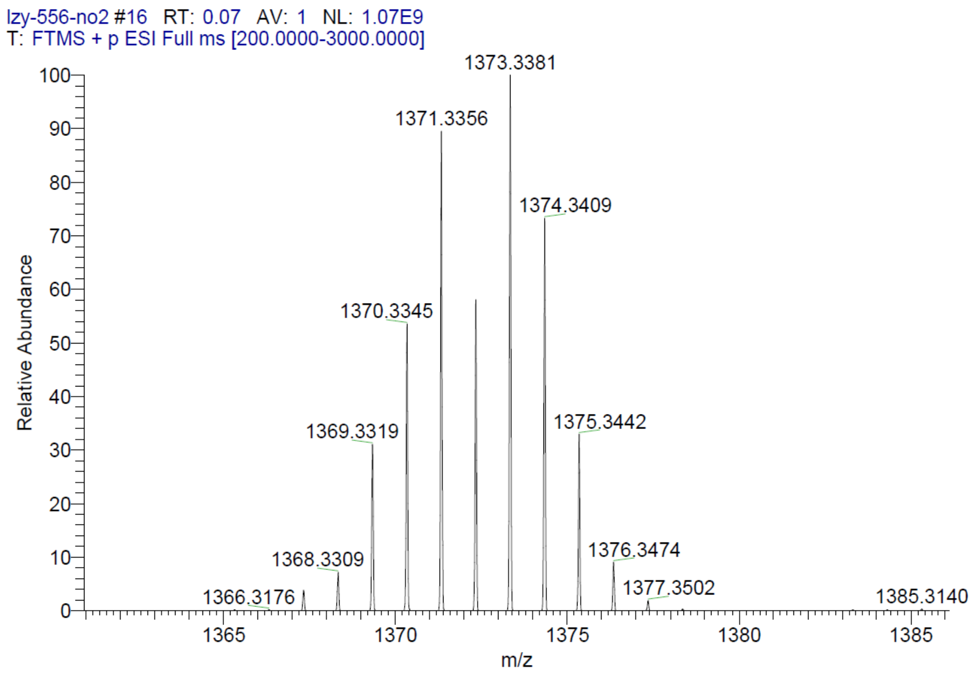

Fig. S12 Positive-ion ESI-MS spectrum of [**3a**]$^+$ measured in methanol

S2.4 Preparation and Characterization of CL-NH

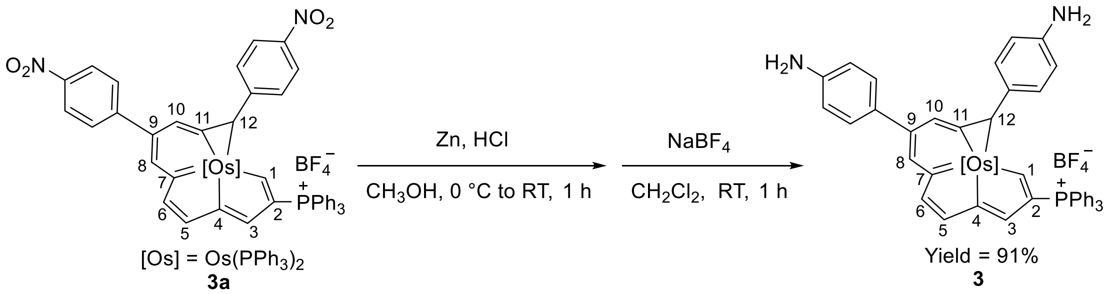

A solution of the complex **3a** (88mg, 0.06 mmol) in methanol (10 mL) and concentrated HCl (0.5 mL) was carefully treated with zinc dust (39 mg, 0.60 mmol) at 0 °C. The suspension was stirred at 0 °C for 30 min and room temperature for 30 min. A saturated aqueous $NaHCO_3$ solution was slowly added. The mixture was extracted using dichloromethane and $NaBF_4$ was added. The mixture was stirred at room temperature for 1 h, and then the solid suspension was removed by filtration. The filtrate was reduced under vacuum to approximately 2 mL, and then loaded on silica gel column eluted by dichloromethane/methanol (20/1). The green band was collected, and the solvent was evaporated to dryness under vacuum. The resultant residue was washed with diethyl ether to give a yellow-green solid of complex CL-NH. Yield, 76 mg, 91%.

^{1}H NMR plus ^{1}H-^{13}C HSQC (400.1 MHz, CD_2Cl_2): δ = 12.86 (d, J_{HH} = 21.4 Hz, 1H, H1), 8.43 (m, 1H, H3), 7.75 (s, 1H, H8), 7.60 (s, 1H, H10), 7.32 (s, 1H, H10), 7.27 (m, 1H, H6), 6.38 (s, 1H, H12), 3.06 (br, 8H, 4H for NH and 4H for H_2O), 7.89–5.75 ppm (53H, other aromatic protons). ^{31}P NMR (162.0 MHz, CD_2Cl_2): δ = 8.81 (t, J_{PP} = 6.0 Hz, C*P*Ph$_3$), -7.16 (dd, J_{PP} = 256.2 Hz, J_{PP} = 6.0 Hz, Os*P*Ph$_3$), -19.54 (dd, J_{PP} = 256.2 Hz, J_{PP} = 6.0 Hz, Os*P*Ph$_3$) ppm. ^{13}C NMR plus DEPT-135, ^{1}H-^{13}C HSQC and ^{1}H-^{13}C HMBC (100.6 MHz, CD_2Cl_2): δ = 231.1 (br, C7), 218.7 (br, C11), 208.7 (br, C1), 200.1 (d, J_{PC} = 27.5 Hz, C4), 162.3 (s, C6), 160.5 (s,C9), 157.8 (s,C5), 135.5 (d, J_{PC} = 27.3 Hz, C3), 125.3 (s, C8), 120.9 (d, J_{PC} = 87.0 Hz, C2), 114.0 (s, C10), 14.5 (s, C12), 149.6–115.2 ppm (other aromatic carbons). ^{11}B NMR (128.4 MHz, CD_2Cl_2): δ = -1.08 (s, *B*F$_4$) ppm. ^{19}F NMR (376.5 MHz, CD_2Cl_2): δ = -153.24 (s, B*F*$_4$) ppm. HRMS (ESI): *m/z* calcd for $[C_{78}H_{64}N_2OsP_3]^+$, 1313.3892; found, 1313.3899.

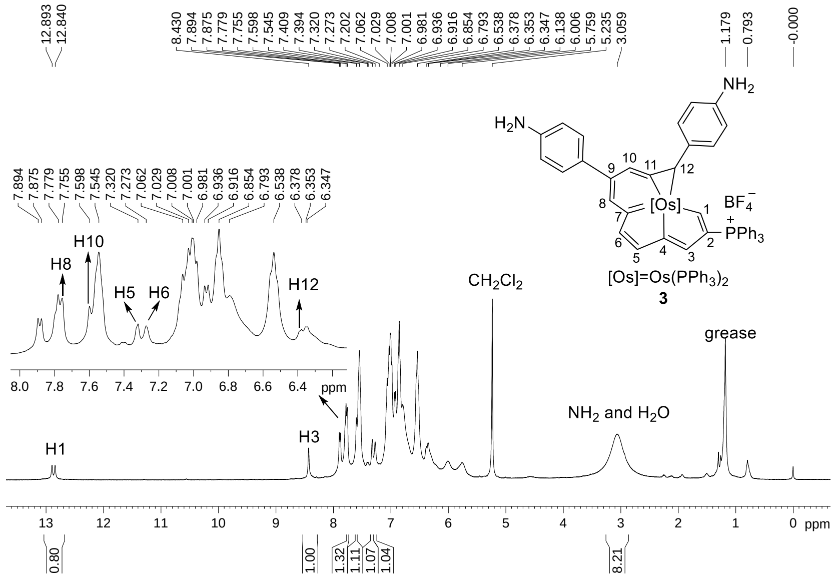

Fig. S13 The ^{1}H NMR (400.1 MHz, CD_2Cl_2) spectrum for complex **CL-NH**

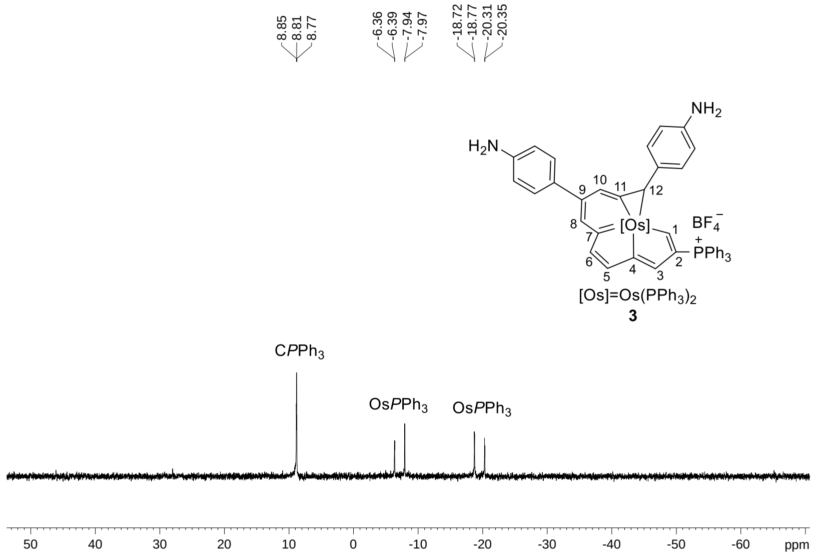

Fig. S14 The ^{31}P{^{1}H} NMR (162.0 MHz, CD_2Cl_2) spectrum for complex **CL-NH**

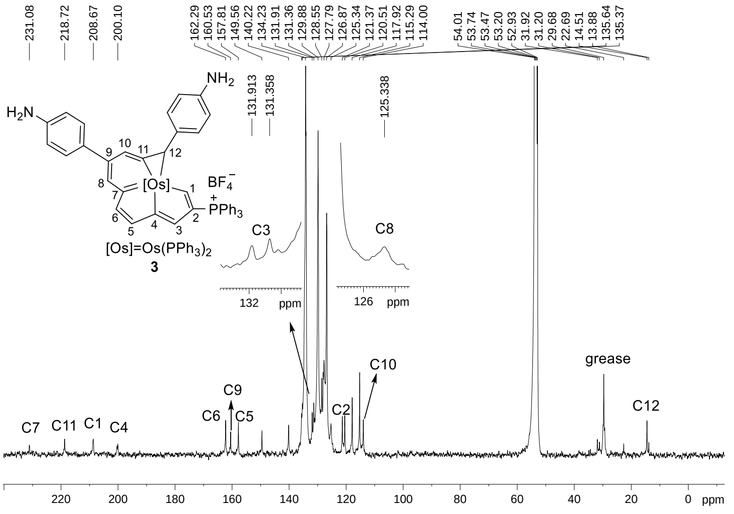

Fig. S15 The ^{13}C{^{1}H} NMR (100.6 MHz, CD_2Cl_2) spectrum for complex **CL-NH**

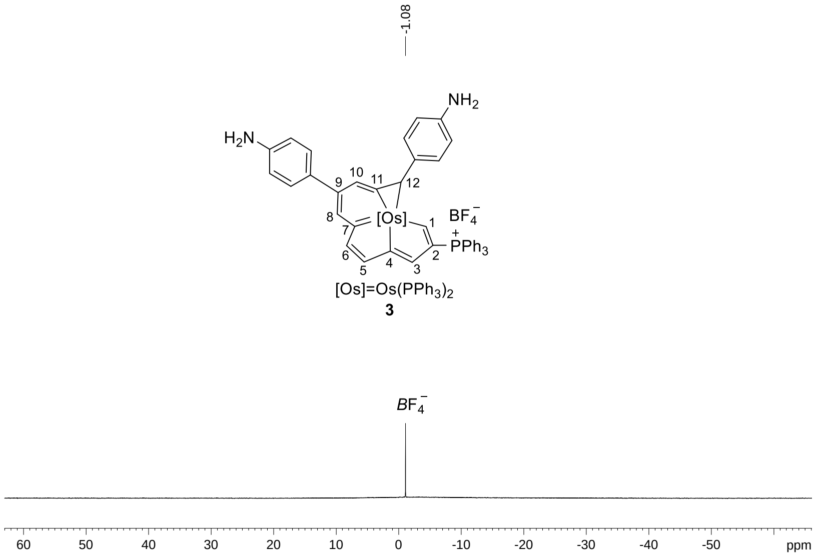

Fig. S16 The ^{11}B{^{1}H} NMR (192.6 MHz, CD_2Cl_2) spectrum for complex **CL-NH**

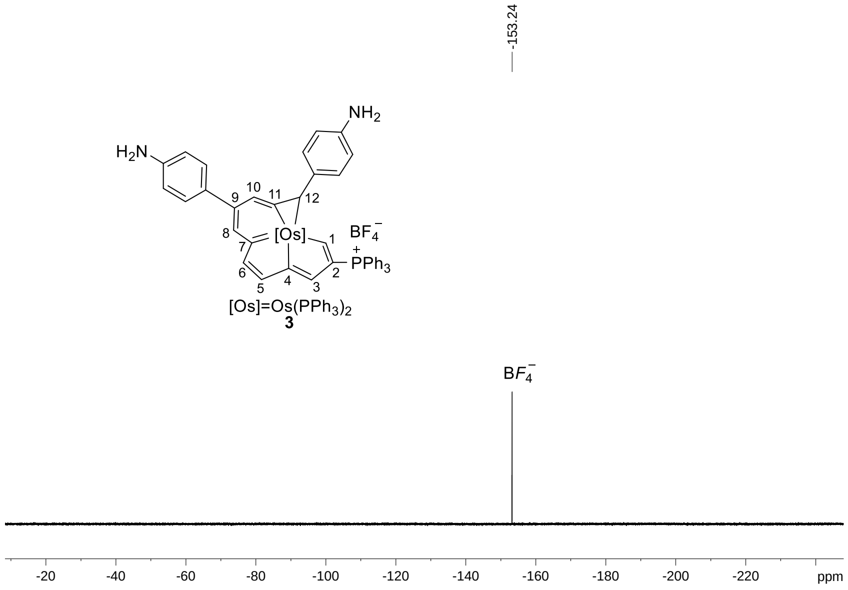

Fig. S17 The ^{19}F{^{1}H} NMR (376.5 MHz, CD_2Cl_2) spectrum for complex **CL-NH**

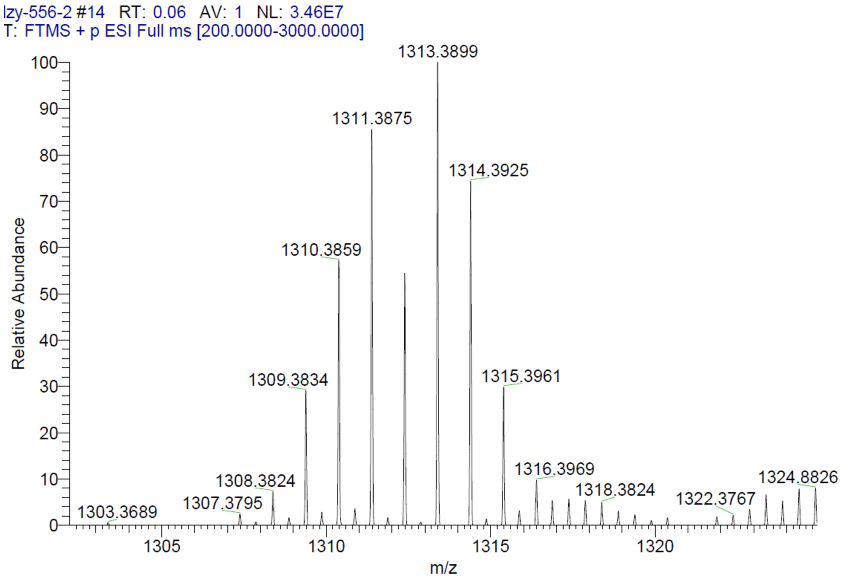

Fig. S18 Positive-ion ESI-MS spectrum of [**CL-NH**]$^+$ measured in methanol

S2.5 Preparation and Characterization of CL-BPh

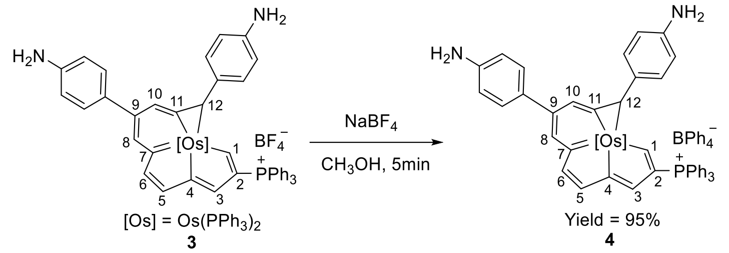

A solution of NaBPh$_4$ (9 mg, 0.026 mmol) in methanol (0.5 mL) was added to a solution of complex **CL-NH** (28 mg, 0.02 mmol) in methanol (2 mL). The reaction mixture was stirred at room temperature for 5 min to give a yellow-green suspension. The solid of complex **CL-BPh** was collected by filtration, washed with methanol and then dried under vacuum. Yield, 31 mg, 95%.

^{1}H NMR (400.2 MHz, CD_2Cl_2): δ = 12.90 (d, J_{HH} = 21.5 Hz, 1H, H1), 4.10 (br, 4H, NH), 8.45–5.77 ppm (80H, other aromatic protons). ^{31}P NMR (162.0 MHz, CD_2Cl_2): δ = 8.70 (br, C*P*Ph$_3$), -7.14 (dd, J_{PP} = 257.1 Hz, J_{PP} = 8.0 Hz, Os*P*Ph$_3$), -19.60 (dd, J_{PP} = 257.1 Hz, J_{PP} = 8.0 Hz, Os*P*Ph$_3$) ppm. ^{11}B NMR (128.4 MHz, CD_2Cl_2): δ = -6.54 (s, *B*Ph$_4$) ppm. HRMS (ESI): *m/z* calcd for $[C_{78}H_{64}N_2OsP_3]^+$, 1313.3892; found, 1313.3894.

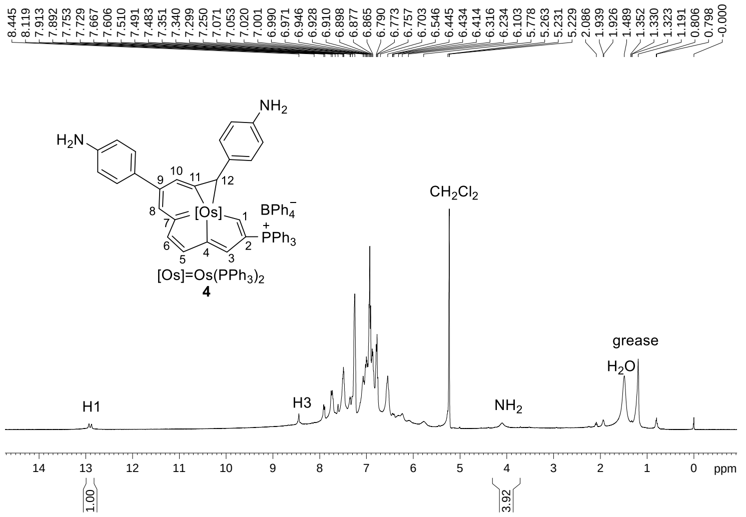

Fig. S19 The ^{1}H NMR (400.2 MHz, CD_2Cl_2) spectrum for complex **CL-BPh**

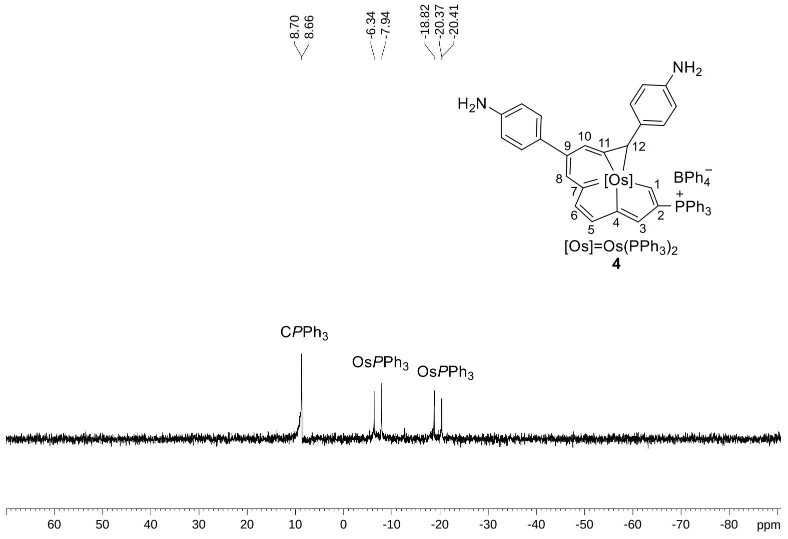

Fig. S20 The $^{31}P\{^{1}H\}$ NMR (162.0 MHz, CD_2Cl_2) spectrum for complex **CL-BPh**

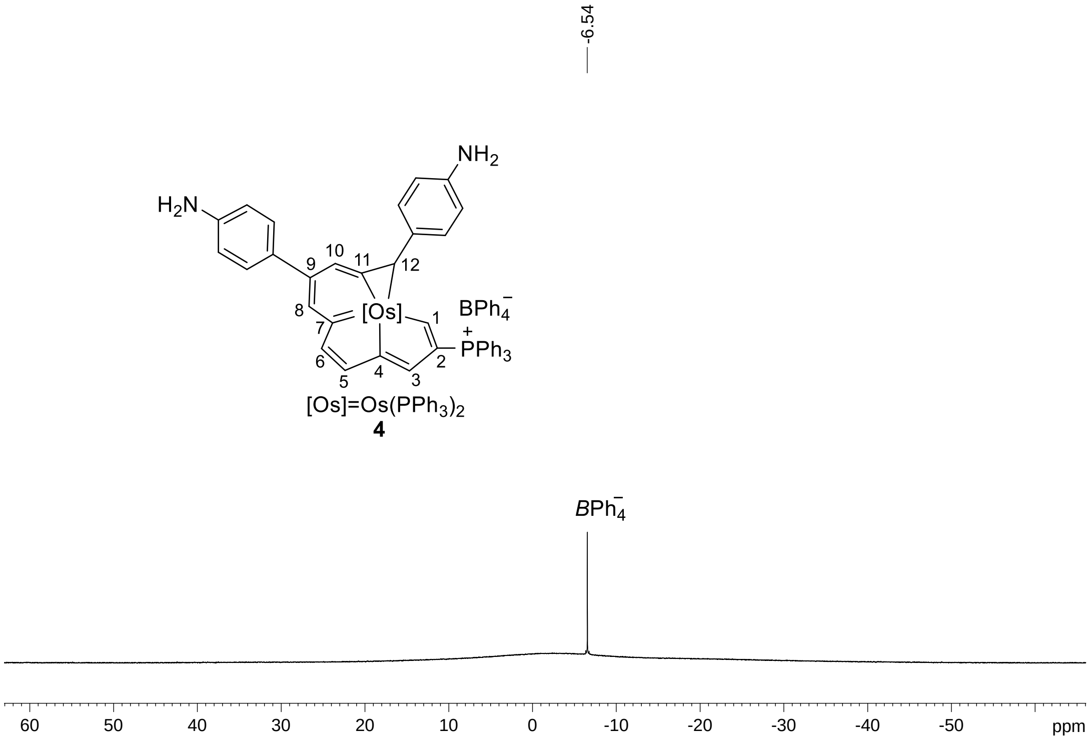

Fig. S21 The $^{11}B\{^{1}H\}$ NMR (192.6 MHz, CD_2Cl_2) spectrum for complex **CL-BPh**

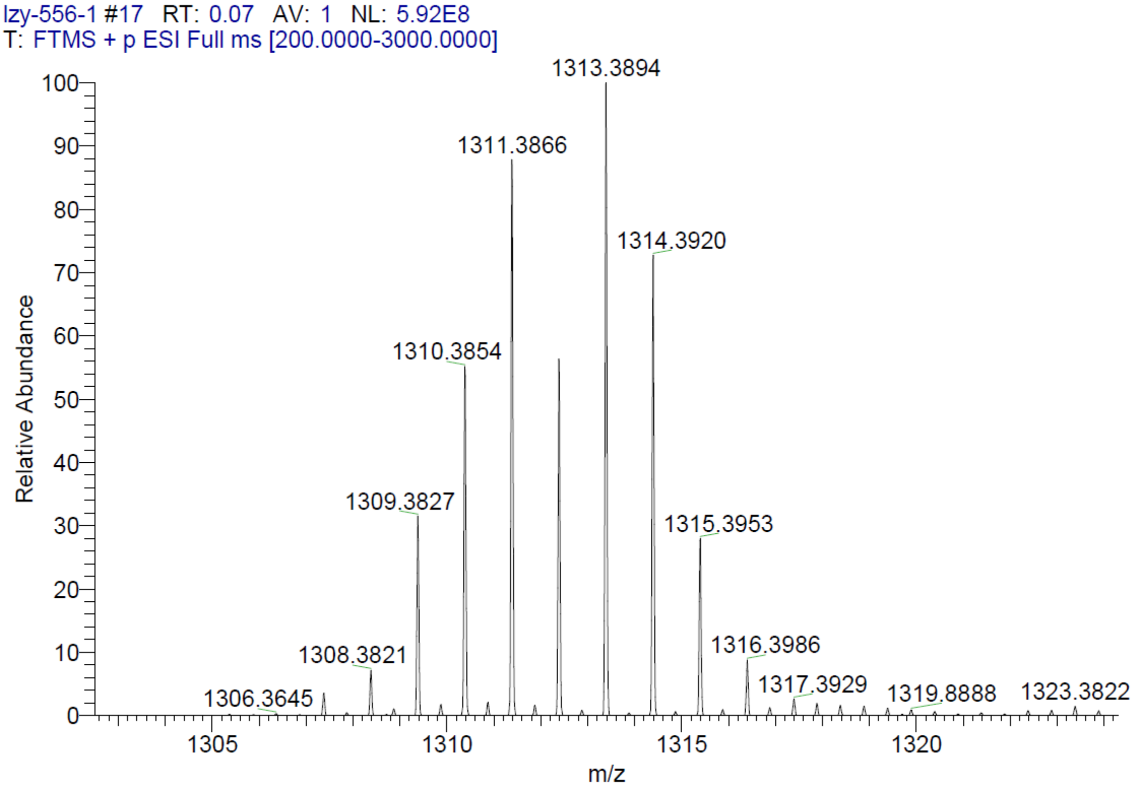

Fig. S22 Positive-ion ESI-MS spectrum of [**CL-BPh**]$^{+}$ measured in methanol

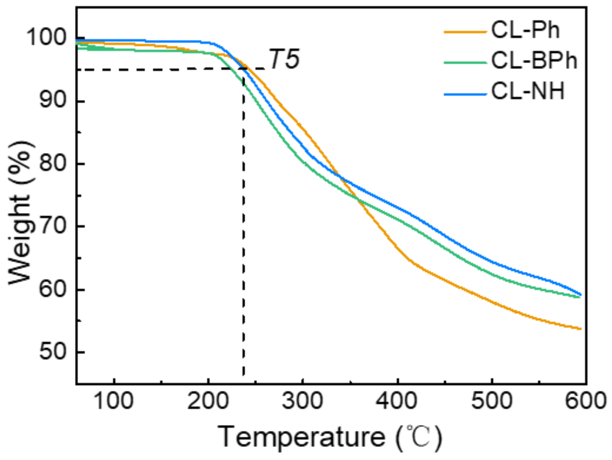

Fig. S23 Thermogravimetric measurements of CL-Ph, CL-BPh and CL-NH complexes

Note: Thermogravimetric analyses (TGA) of CL-Ph, CL-BPh and CL-NH show that the initial decomposition temperatures (T5) as measured at the point of 5% weight loss are 242.9 °C, 224.1 °C and 237.8 °C, respectively.

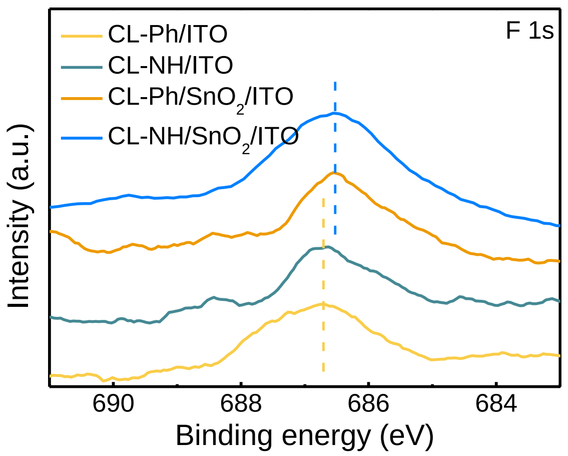

Fig. S24 a) XPS F 1s core spectra of CL-Ph/ITO, CL-NH/ITO and CL-Ph/ITO/SnO_2, CL-NH/ITO/SnO_2, respectively

Note: This energy shift is mainly ascribed to the interaction between high electron negative F atoms in BF_4^- anion and SnO_2 films, which is further verified by the lower energy shift of F 1s spectra.

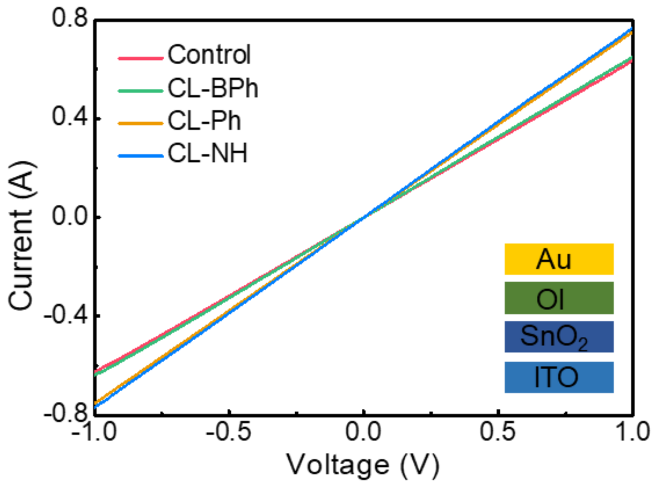

Fig. S25 Conductivity of bare SnO_2, CL-Ph-modified SnO_2, CL-BPh-modified SnO_2 and CL-NH-modified SnO_2, respectively

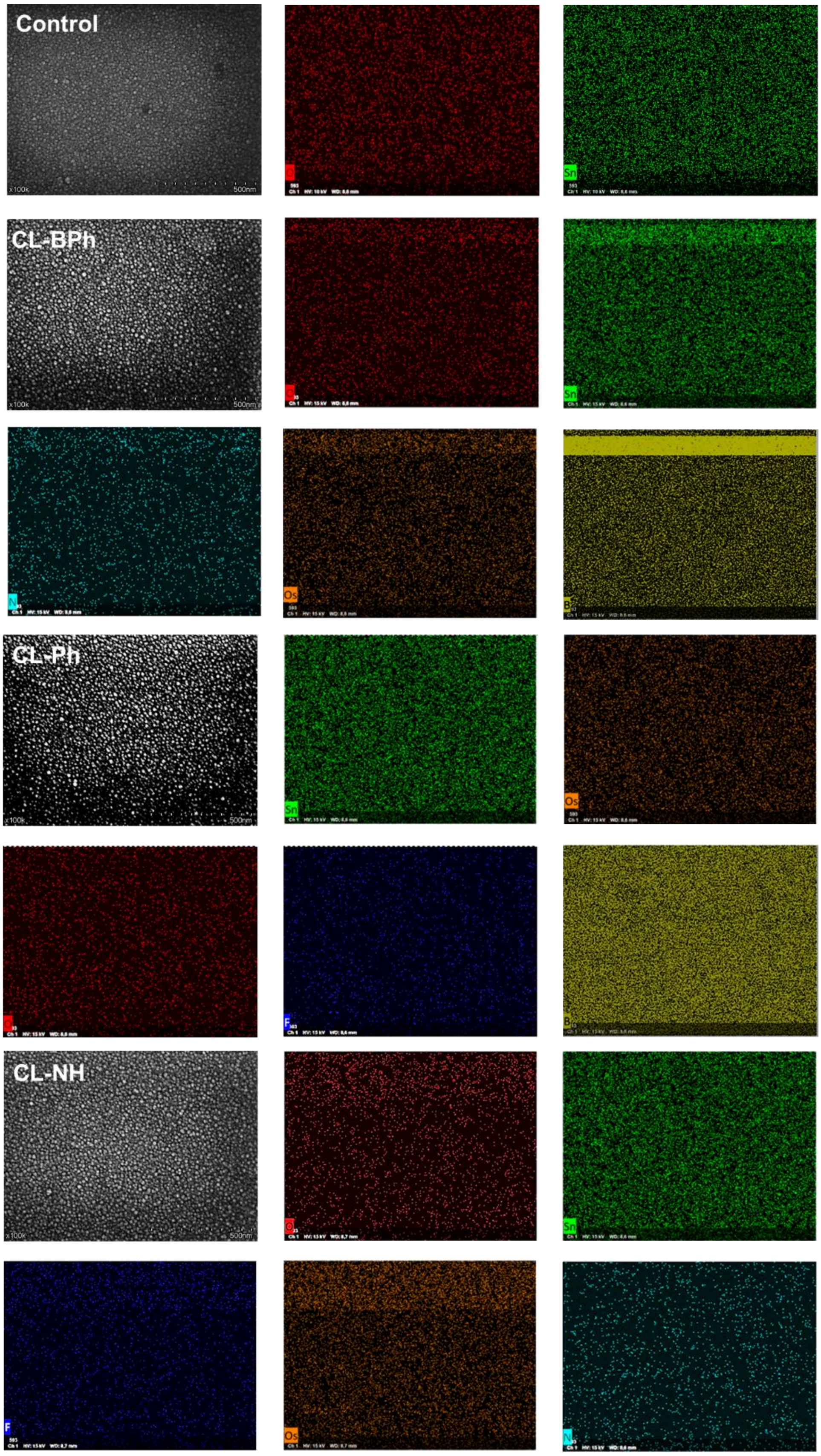

Fig. S26 SEM and element mapping analysis of SnO_2, SnO_2/CL-BPh, SnO_2/CL-Ph anf SnO_2/CL-NH film

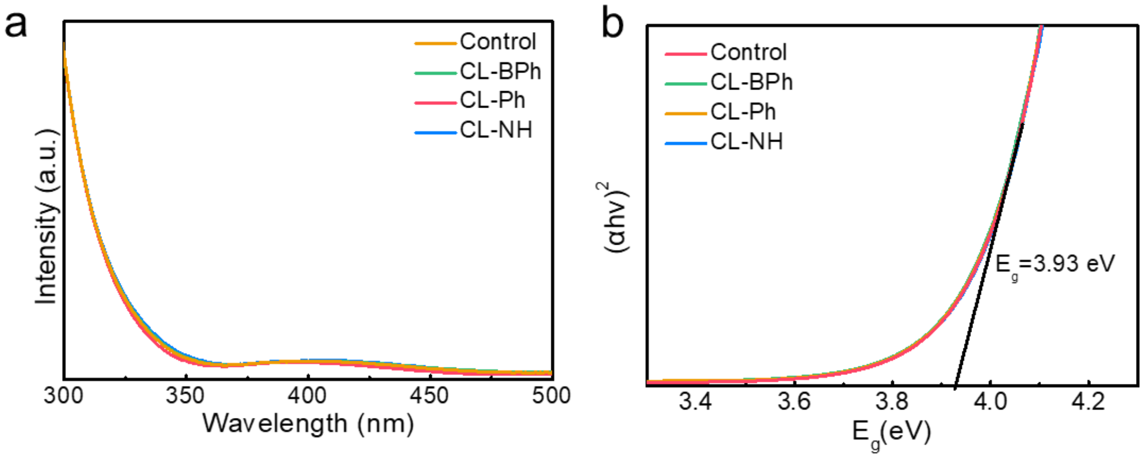

Fig. S27 a) Absorption spectra of SnO_2, SnO_2/CL-BPh, SnO_2/CL-Ph and SnO_2/CL-NH. **b**) Corresponding Tauc-plots for bandgap extraction

Note: The UV–vis spectrum implies that the presence of CL-Ph, CL-BPh , CL-NH molecules does not affect the bandgap of the SnO_2.

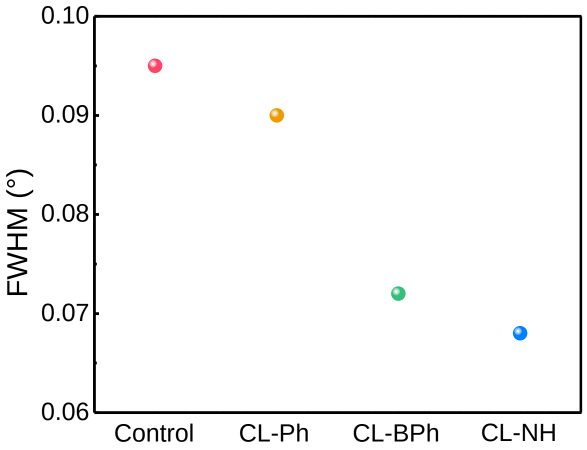

Fig. S28 FWHM of main peaks between 13.9° and 14.9°for the Control, CL-Ph, CL-BPh and CL-NH perovskite films

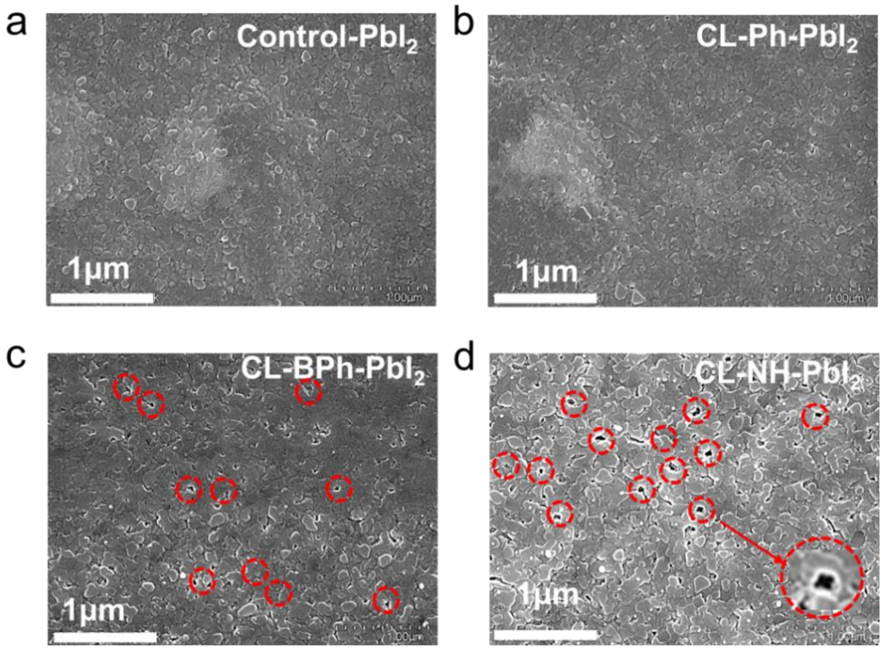

Fig. S29 SEM of PbI_2, CL-Ph-PbI_2, CL-BPh-PbI_2 and CL-NH-PbI_2film

Note: As shown in Fig. S29, no pinholes can be observed from the control and CL-Ph films. Only some valleys between adjacent crystals, as marked in white, can be observed. However, for the CL-BPh and CL-NH films (Fig. S29c,d), there are some pinholes (marked in red), which are totally different from the adjacent crystal valleys, as supported by the insert enlarged images, can be clearly observed. These pinholes can facilitate the penetration of organic salt, which further improve the crystallinity of the resulting perovskite film.

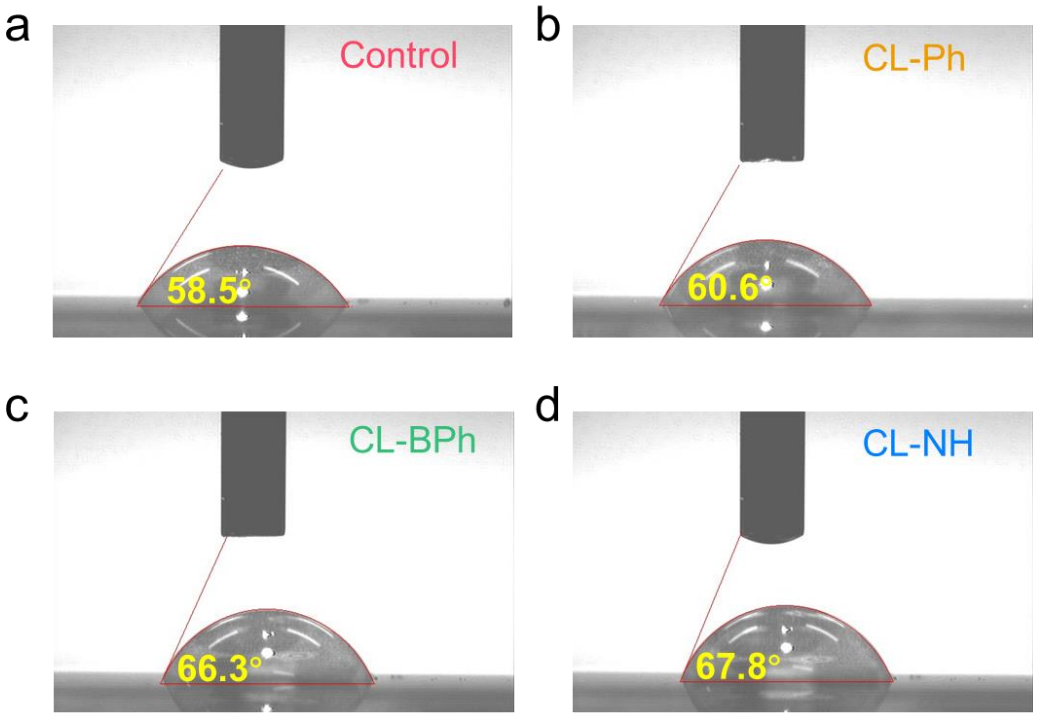

Fig. S30 Water contact angle results of different perovskite films

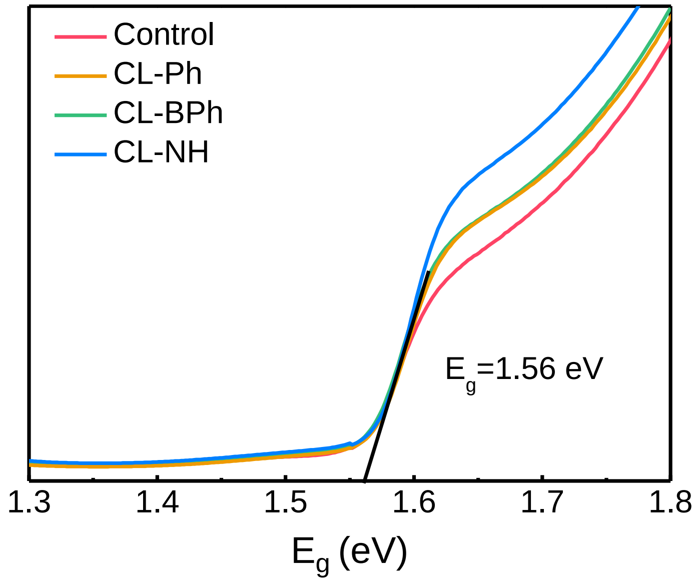

Fig. S31 Tauc's plot calculated from the UV-Vis absorption spectra with equation $(\alpha h\upsilon)^2 = A(h\upsilon - E_g)$, where α is the absorption coefficient, hυ is the photon energy, and E_g is the bandgap

Note: The UV–vis spectrum implies that the presence of CL-Ph, CL-BPh , CL-NH molecules does not affect the bandgap of the perovskite materials. However, the existence of CL-Ph, CL-BPh , CL-NH molecules makes the UV-Vis absorption intensity enhanced.

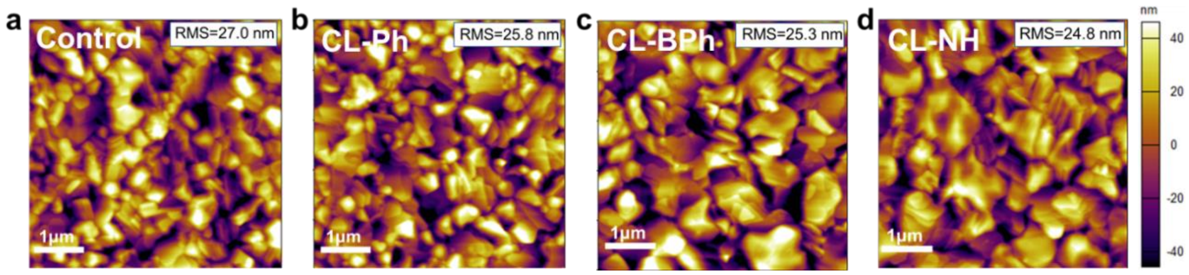

Fig. S32 AFM image of control, CL-Ph, CL-BPh and CL-NH perovskite films

Note: The OI complexes treated films reveal larger perovskite grains and smaller root mean square (RMS) values, which is helpful for charge extraction.

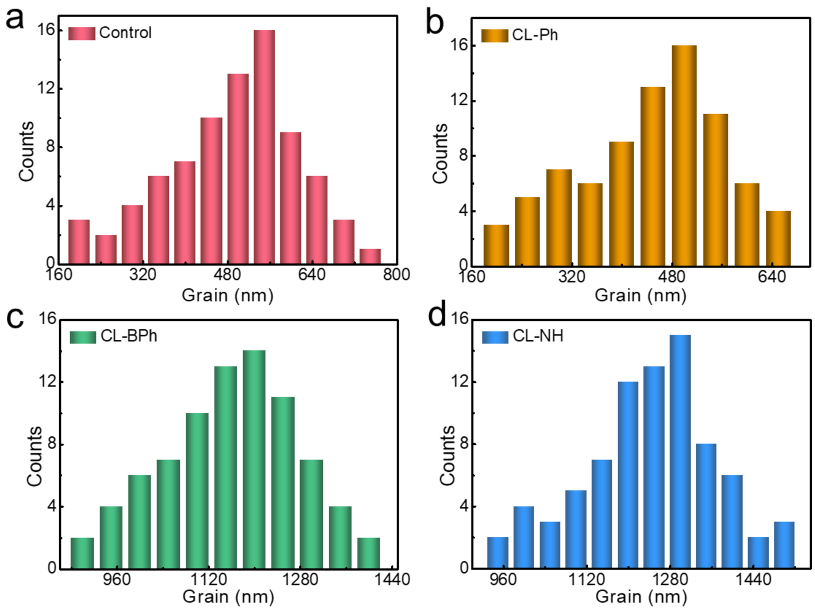

Fig. S33 Grain size statistics for Control, CL-Ph, CL-BPh and CL-NH perovskite films

It is noted that the control and CL-Ph films show an average grain size at around 500 nm, while, the CL-BPh and CL-NH films show much larger grain size at around 1150 nm. The significant enhancement on grain size after CL-BPh and CL-NH modification is mainly attributed to the improvement of film crystallinity due to the interaction between -NH_2 group and PbI_2.

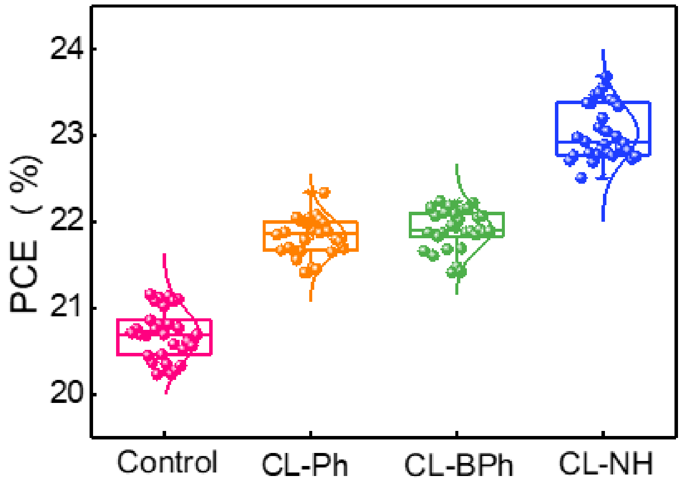

Fig. S34 Statistical PCE parameters of 30 independent devices based on control, CL-Ph, CL-BPh and CL-NH films

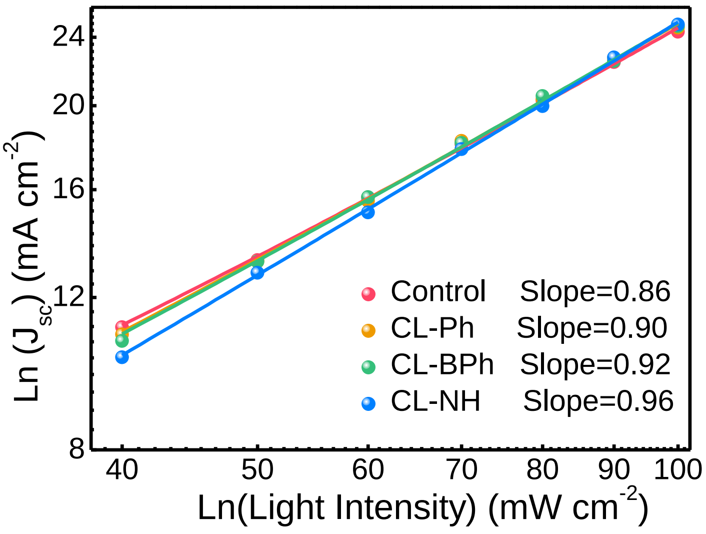

Fig. S35 Light-intensity dependence of $J_{sc}$of PSCs devices fabricated by control and CL-Ph, CL-BPh, CL-NH complexes modified SnO_2 films

Note: The J_{sc} versus light intensity of the devices reveal a linear relationship, indicating a favorable environment for charge extraction. While, the most ideal α value of 0.96 for CL-NH device suggests the formation of high-quality perovskite film with better aligned energy level at ETL/perovskite interface, thus facilitating charge extraction and collection.

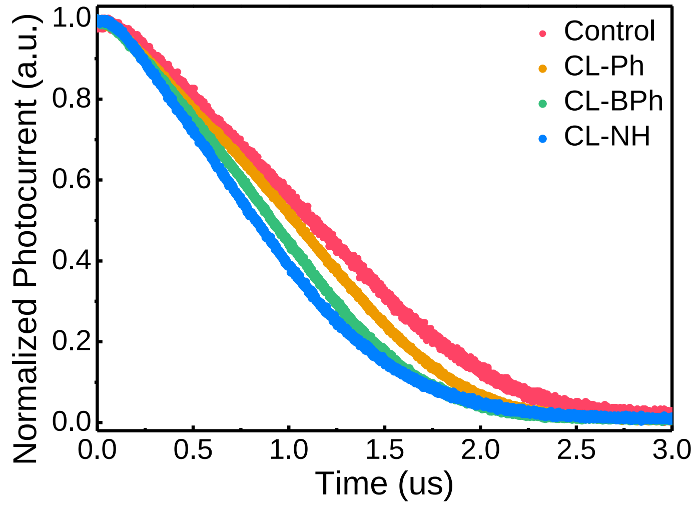

Fig. S36 Transient photocurrent decay of PSCs devices fabricated by control and CL-Ph, CL-BPh, CL-NH complexes modified SnO_2 films

Note: According to the TPC results, the fastest charge extraction time 0.84 us derived from CL-NH device indicates an enhanced charge extraction efficiency, which is responsible for the highest J_{sc}, and detail parameters summarized in Table S5.

Table S1 By the Figure 1e (XPS high-resolution spectra of SnO_2 and SnO_2/ CL-Ph, SnO_2/ CL-BPh, SnO_2/ CL-NH for O 1s.) calculating the peak areas

Sample	O_V+O_{OH}	Area	Sn-O	Area
Control	531.61 eV	40.2 %	530.31 eV	59.8 %
CL-BPh	531.64 eV	45.2 %	530.34 eV	54.8 %
CL-Ph	531.96 eV	29.6 %	530.66 eV	70.4%
CL-NH	531.98 eV	24.9 %	530.68 eV	75.1%

Table S2 Conduction band minimum (CBM) and valence band maximum (VBM) of control and CL-Ph, CL-BPh, CL-NH complexes modified SnO_2 films

SnO_2	VBM	CBM	E_g	WF
Control	8.32	4.39	3.93	4.70
CL-Ph	8.27	4.34	3.93	4.60
CL-BPh	8.31	4.38	3.93	4.68
CL-NH	8.25	4.32	3.93	4.54

Table S3 Photovoltaic performance of the champion devices for the corresponding PSCs

Device	Scan direction	V_{oc} [V]	J_{sc} [mA cm^{-2}]	FF [%]	PCE [%]	Integrated J_{sc} [mA cm^{-2}]
Control	Reverse	1.133	24.63	75.70	21.13	23.61
CL-Ph	Reverse	1.166	24.92	75.93	22.08	23.98
CL-BPh	Reverse	1,167	24.58	77.49	22.24	23.87
CL-NH	Reverse	1.187	25.22	79.12	23.69	24.14

Table S4 TRPL parameters of the control, CL-Ph, CL-BPh, CL-NH complexes modified SnO_2 films

Sample	A_1	τ_1 [ns]	A_2	τ_2 [ns]	τ_{ave} [ns]
Control	4.41	31.25	0.56	414.99	271.54
CL-Ph	0.58	19.86	0.57	226.82	207.96
CL-BPh	0.49	36.61	0.55	191.91	168.81
CL-NH	0.68	12.49	0.53	137.69	124.41

Note: The fitting biexponential decay function was $y = y_0 + A_1 exp\left(-\frac{t}{\tau_1}\right) + A_2 exp\left(-\frac{t}{\tau_2}\right)$, $\tau_{ave}= \frac{A_1 t_1^2 + A_2 t_2^2}{A_1 t_1 + A_2 t_2}$.

Supplementary References

[S1] C. Zhu, X. Zhou, H. Xing, K. An, J. Zhu et al., σ-aromaticity in an unsaturated ring: osmapentalene derivatives containing a metallacyclopropene unit. Angew. Chem. Int. Ed. **54**, 3102-3106 (2015). https://doi.org/10.1002/anie.201411220

[S2] C. Zhu, C. Yang, Y. Wang, G. Lin, Y. Yang et al., CCCCC pentadentate chelates with planar Möbius aromaticity and unique properties. Sci. Adv. **2**, e1601031 (2016). https://doi.org/10.1126/sciadv.1601031

[S3] X. Lin, W. Xie, Q. Lin, Y. Cai, Y. Hua et al., NIR-responsive metal-containing polymer hydrogel for light-controlled microvalve. Polym. Chem. **12**, 3375-3382 (2021). https://doi.org/10.1039/D1PY00404B
